# Pharmaceutical and Recreational Drug Usage Patterns during and Post COVID-19 Determined by Wastewater-Based Epidemiology

**DOI:** 10.3390/ijerph21020206

**Published:** 2024-02-09

**Authors:** Laura Elina Tomsone, Romans Neilands, Kristina Kokina, Vadims Bartkevics, Iveta Pugajeva

**Affiliations:** 1 Institute of Food Safety, Animal Health and Environment “BIOR”, Lejupes Street 3, LV-1076 Riga, Latvia; laura.tomsone@bior.lv (L.E.T.);; 2 Faculty of Natural Sciences and Technology, Riga Technical University, Kipsalas Street 6B, LV-1048 Riga, Latvia

**Keywords:** wastewater-based epidemiology, caffeine, alcohol, nicotine, pharmaceuticals, COVID-19

## Abstract

Wastewater-based epidemiology (WBE) was applied to evaluate the consumption trends of pharmaceuticals (i.e., antibiotics, non-steroidal anti-inflammatory drugs, antiepileptics, antihypertensives, and others), as well as recreational drugs (caffeine, alcohol, and nicotine), in Latvia from December 2020 to July 2023. The time period covers both the COVID-19 pandemic and the post-pandemic periods; therefore, the impact of the implemented restrictions and the consequences of the illness in terms of the usage of pharmaceuticals thereon were investigated. Additionally, the seasonality and impact of the seasonal flu and other acute upper respiratory infections were studied. The results revealed that the pandemic impacted the consumption of alcohol, nicotine, and caffeine, as well as several pharmaceuticals, such as antihypertensives, antidepressants, psychiatric drugs, and the painkiller ibuprofen. The findings suggest that the imposed restrictions during the pandemic may have had a negative effect on the population’s health and mental well-being. Distinct seasonal trends were discovered in the consumption patterns of caffeine and alcohol, where lower use was observed during the summer. The seasonal consumption trends of pharmaceuticals were discovered in the case of antibiotics, the antiasthmatic drug salbutamol, and the decongestant xylometazoline, where higher consumption occurred during colder seasons.

## 1. Introduction

Wastewater-based epidemiology (WBE) is an effective tool that uses determined concentrations in raw wastewaters to provide information on human consumption and exposure to chemical residues at a community level. Commonly analysed compounds include illicit drugs, food ingredients, alcohol and tobacco, pharmaceuticals, pesticides, personal care products, and various pollutants [1,2,3,4]. Recently, WBE has also proven to be a valuable tool for the rapid and cost-effective determination of SARS-CoV-2 [5].

WBE involves systematic sampling of wastewater at the influent of wastewater treatment plants (WWTPs) and quantitative determination of analytes of interest therein. Measured concentrations of analytes are translated into consumption data by correcting for compound-specific excretion factors and normalising to the size of the contributing population in the catchment area of WWTPs [6].

Generally, wastewater composition reflects society’s habits. WBE provides insights into population lifestyle, health, and behaviour. Recent studies have shown that there is a link between WBE results and sociodemographic and socioeconomic descriptors and changes [7,8]. WBE has been widely applied to studies of spatial and temporal trends in illicit drug use [3,9]. It can be employed to obtain helpful information on the consumption patterns of pharmaceuticals over time. For instance, long-term trends (i.e., trends that occur over several months or years) can indicate whether pharmaceutical use is stable, fluctuating, declining, or rising. In contrast to illicit drugs, short-term (i.e., within a week or month) variations in pharmaceutical consumption are less expected since pharmaceutical treatment requires frequent dose intervals and fixed treatment schemes. For this reason, it is more interesting to monitor long-term consumption patterns in the use of pharmaceuticals [10].

For the past few years, our society has been facing the consequences of COVID-19, which has had a serious impact on health, social, economic, and behavioural aspects [11]. To fight the disease, high consumption of antivirals, antibiotics, antiparasitics, antiprotozoals, and glucocorticoids used in the treatment of this virus has been reported [12,13,14]. It has also been reported that for some pharmaceuticals, a decline in use has been observed, probably due to difficulty reaching healthcare facilities or visiting physicians to refill prescriptions [15]. The pandemic also caused a multitude of interventions, such as nationwide lockdowns, school closures, cancellation of public events, travel restrictions, and curfews. These restrictions have been shown to cause changes in recreational drug consumption (caffeine, alcohol, nicotine) [3,16]. Moreover, the COVID-19 crisis has been shown to affect mental health, leading to increased use of alcohol, nicotine, and antidepressants [17,18,19].

Changes in the consumption of pharmaceuticals and recreational drugs during the COVID-19 pandemic have been clearly observed by other WBE studies; however, most of them cover only a period of one year or compare differences between two years. Such studies might be susceptible to random uncertainties and errors due to a lack of reference data, as identified by Perkons et al. [9]. To our knowledge, only a few studies present long-term monitoring covering multiple years [3,14].

This work uses WBE tools to identify the direct and indirect effects of the recent crisis on public health. In particular, this study aims to identify trends in the use patterns of pharmaceutics and recreational drugs during the COVID-19 lockdown in Riga, the capital of Latvia. In order to achieve the aforementioned objective, the most popular recreational drugs, such as alcohol, nicotine, and caffeine, as well as representatives of the classes of antibiotics, non-steroidal anti-inflammatory drugs, antiepileptics, antihypertensives, antihyperlipidemics, analgesics, antivirals, psychiatrics, and antidiabetics were investigated. The concentrations of 40 markers were determined in wastewater collected every week at the influent of Riga main WWTP during the COVID-19 lockdown and after (from 22 December 2020 to 4 July 2023). Concentrations were converted to consumption data to identify temporal trends during the monitoring campaign. To our knowledge, this is one of the first studies covering multiple years and comprising a wide list of pharmaceuticals as well as the most popular recreational drugs.

## 2. Experimental

### 2.1. Chemicals and Materials

Acetic acid (99.7%) was purchased from Sigma-Aldrich (St. Louis, MO, USA). Ultrafree-MC centrifugal filters 0.22 μm pore size, hydrophilic PVDF, and HPLC-gradient-grade methanol were purchased from Merck (Darmstadt, Germany). Strata-X SPE cartridges and chromatographic column were obtained from Phenomenex (Torrance, CA, USA). The water was deionized by a Milli-Q water purification system (Millipore, Billerica, MA, USA).

All analytical standards used in the current study were purchased from LGC standards (Lomianki, Poland), Sigma-Aldrich (St. Louis, MO, USA), or Fluka (Buchs, Switzerland).

### 2.2. Selection of Analytes

For the current study, we selected 40 compounds. These were chosen based on data collected during the first sampling campaign [20]. We focused on pharmaceutical substances and included popular recreational drugs, such as alcohol (determined as ethyl sulphate), nicotine (determined as cotinine), and caffeine, as lifestyle biomarkers. For normalisation of selected drug concentrations, endogenous compound 5-HIAA was measured across different population sizes and wastewater flow rates. It has been proven to be stable in sewers and suitable to be measured using simple and rapid analytical methods [21,22].

### 2.3. Collection of Samples

Sampling was carried out from December 2020 to July 2023 at a wastewater treatment plant (WWTP) located in Riga, Latvia. This plant has a capacity of 200,000 m^3^ per day and caters to 697,000 inhabitants. An automated sampler (ASP-Station 2000, vacuum system RPS20, Endress+Hauser, Greenwood, IN, USA) was used to collect each flow-proportional composite sample of influent wastewater over a 24 h period. Sampling was conducted every week from Monday morning to Tuesday morning throughout the entire sampling period, resulting in a total of 132 samples. These samples were collected in 1.5 L polyethylene terephthalate bottles, immediately frozen, and stored at −20 °C until analysis.

### 2.4. Sample Preparation and Instrumental Analysis

The samples were analysed within four weeks of collection to prevent chemical degradation. The analysis of the sample was carried out using the procedure previously described by Pugajeva et al. [23]. In short, two protocols were used to prepare each sample, namely solid-phase extraction (SPE) on Strata-X cartridges (200 mg/3 mL) and dilute and shoot by diluting 2 mL of the unfiltered sample with 8 mL of deionized water and 10 µL of formic acid. The analysis was performed using heart-cutting two-dimensional high-performance liquid chromatography (2D-HPLC) separation of selected biomarkers using a Kinetex^®^ C18 column (1.7 μm, 100 Å, 50 mm × 3 mm; Phenomenex, Torrance, CA, USA) for the first chromatographic separation and a Synergy^TM^ Max-RP column (4 μm, 80 Å, 150 × 3 mm; Phenomenex, Torrance, CA, USA) for the second dimension. The HPLC system was coupled to a triple-quadrupole mass spectrometer (ThermoFisher Scientific TSQ Altis^TM^, San Jose, CA, USA) equipped with a heated electrospray ionization probe (HESI). The sample analysis was conducted in the selected reaction monitoring mode by scanning two ion transitions for each compound. The following HESI parameters were applied: spray voltage 4.0 kV, vaporizer temperature 400 °C, ion transfer tube temperature 300 °C, sheath gas (N_2_) flow 40 arbitrary units (arb), and auxiliary gas (N_2_) flow 10 arb. Analytes were quantified using multiple-level procedural calibration in deionized water. Salbutamol-d3 and ibuprofen-d3 were used as internal standards to compensate for matrix effects during LC-MS/MS analyses.

The validation of the method was carried out on deionized water because there was no wastewater available that was free of the selected analytes. Both sample preparation methods showed good linearity, with determination coefficients greater than 0.990 for all substances within the selected concentration ranges of 10–500 ng L^−1^ and 1–200 µg L^−1^. The mean repeatability of the test results varied from 2.5% to 31%, while the trueness ranged from 69% to 143%. The limit of quantification (LOQ) was determined experimentally as the lowest concentration of an analyte in the sample for which the S/N ratio exceeded 10. The LOQ values varied from 0.1 to 50 ng L^−1^ and from 0.5 to 5 µg L^−1^ for the SPE and dilution methods, respectively. The validation data can be found in an article by Pugajeva et al. [23].

During sample analysis, quality assurance was ensured by using fortified wastewater samples to account for matrix differences. To quantify the samples, calibration samples were prepared in deionized water. Within each batch, several quality control samples were analysed, and the concentrations of the analytes detected in the samples were adjusted using the average recovery values that were obtained for each compound within that batch of samples. Additionally, at least two samples from different batches were reanalysed with subsequent batches to verify data consistency. As an acceptance criterion, the relative standard deviation of the replicates should have been below 20% or the reproducibility value obtained during the validation of corresponding compounds.

### 2.5. Consumption Calculation

Estimating consumption through back-calculation from biomarker concentrations is a major challenge in WBE. A variety of parameters must be considered (population, flow rates, pharmacokinetic data, in sewer and storage stability) [24]. For this study, we used the population biomarker approach to reduce uncertainty in estimating the population size and daily wastewater treatment plant (WWTP) flow rate [21]. The values used for calculations are summarized in Table S1 in the Supporting Information. Consumption of selected compounds was calculated using Equation (1):

(1) $$Consumption=\frac{C_{marker}\cdot C_{f}\cdot E_{r\left(HIAA\right)}}{C_{HIAA}{\cdot E}_{f\left(marker\right)}}$$

where consumption was measured in mg inh^−1^ day^−1^, C_marker_ (ng L^−1^) was the concentration of the biomarker in untreated wastewater, C_HIAA_ (ng L^−1^) was the concentration of population biomarker 5-HIAA in untreated wastewater, and C_f_ was the correction factor obtained considering the molecular mass ratio of parent drug and measured biomarker (where applicable). Alcohol consumption was expressed in mL inh^−1^ day^−1^ by applying the density factor of ethanol (*ρ* = 0.789 g mL^−1^). The average daily excretion rate of 5-HIAA (E_r(HIAA)_) was 4.16 mg inh^−1^ day^−1^ [21]. E_f_ was the excretion factor of the selected biomarker.

In order to express drug consumption as defined daily doses (DDDs) per 1000 inhabitants per day, pharmaceutical consumption was calculated using Equation (2).

(2) $${Consumption}_{pharm}=\frac{Consumption}{DDD}\cdot 1000$$

In order to express the consumption of alcohol and nicotine among the population aged 15 years or older, which is 84.1% of the total population in Latvia [25], Equation (3) was applied.

(3) $${Consumption}_{15+}=\frac{Consumption}{0.841}$$

## 3. Results and Discussion

In this study, a continuous wastewater sampling campaign of 32 months was conducted in order to evaluate the consumption trends for various pharmaceuticals and recreational drugs. Long-term WBE studies provide valuable insight into drug consumption patterns and are less affected by short-term fluctuations. This research is an extension of the previously reported year-long monitoring campaign covering 2021 [20]. In the first stage of the study, consumption trends during the COVID-19 pandemic were observed; however, the lack of reference data obtained during the third year of the pandemic, when many restrictions were cancelled (e.g., in May 2022, the requirement to wear masks in public transportation in Latvia was repealed), might have obscured the interpretation of the findings.

### 3.1. Consumption Estimation

**Figure 1:**
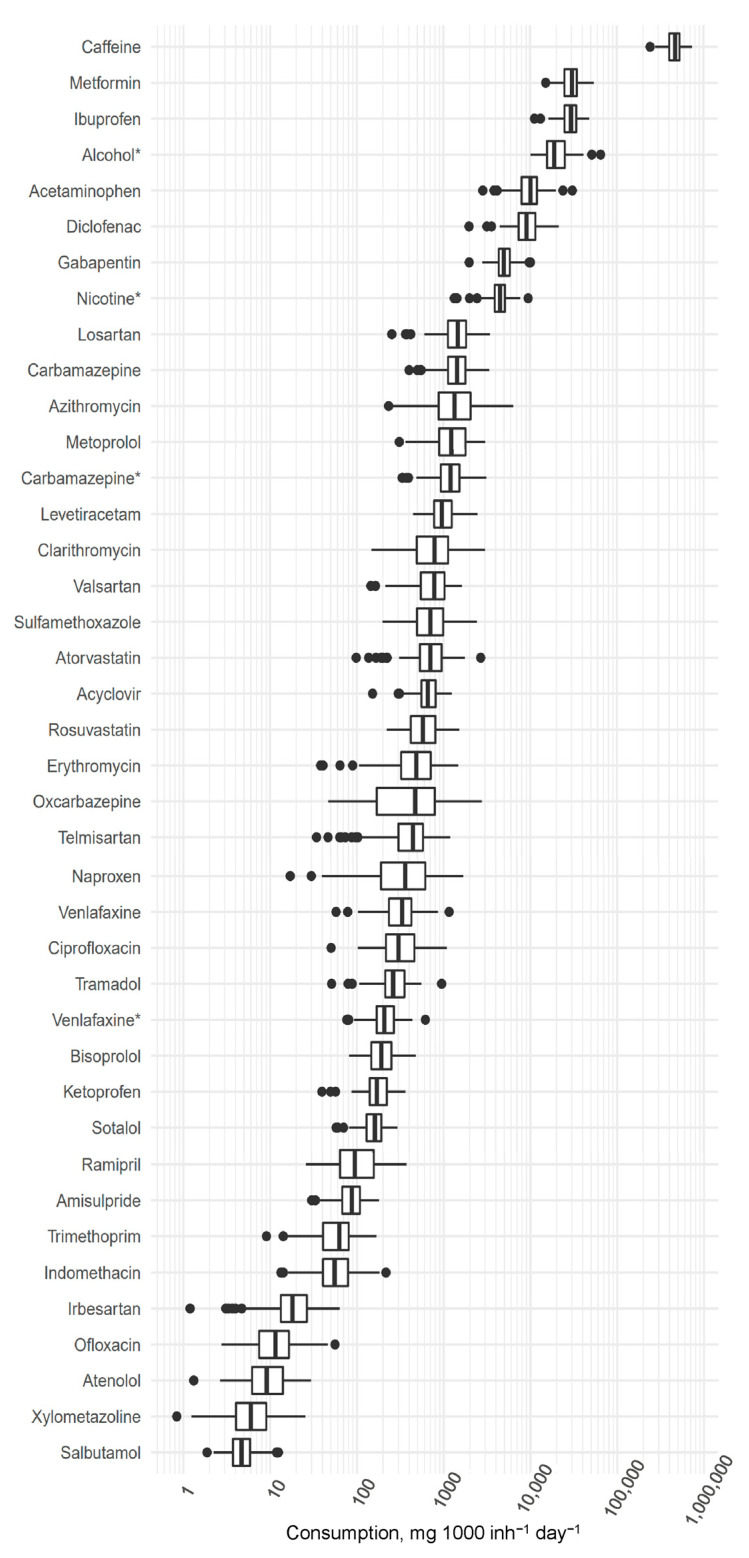
Consumption values obtained for the 40 targeted compounds between December 2020 and July 2023 (asterisk marks compounds that were determined as metabolites).

An overview of the ranges of the calculated compound-specific consumption rates obtained during the whole study period is given in Figure 1, depicted as a box plot, showing the distribution of data in terms of median, first and third quartile (box), minimum, maximum values (whiskers), and outliers (marked as points). Detailed information is available in Supporting Information Table S2. For uniformity purposes, the consumption of all compounds is expressed in mg 1000 inh^−1^ day^−1^. All 40 targeted compounds were detected in each composite wastewater sample. It should be noted that in this dataset, carbamazepine and venlafaxine each have two consumption values reported—one calculated from the parent compound and the other from metabolites (carbamazepine-10,11-epoxide and O-desmethylvenlafaxine, respectively). The obtained consumption ranges spanned 6 orders of magnitude. Caffeine was the most abundant compound analysed—an average value of 470,671 mg 1000 inh^−1^ day^−1^ was obtained, followed by the antidiabetic drug metformin at 30,414 mg 1000 inh^−1^ day^−1^, the pain medication ibuprofen at 29,731 mg 1000 inh^−1^ day^−1^, and alcohol at 21,100 mg 1000 inh^−1^ day^−1^. The lowest consumption rates were observed in the case of the anti-asthmatic drug salbutamol, 5.25 mg 1000 inh^−1^ day^−1^, and the decongestant xylometazoline, 7.06 mg 1000 inh^−1^ day^−1^.

The variability in consumption rates during the reference period can be expressed by relative standard deviations (RSDs). The RSDs ranged from 21% for caffeine to 85% for oxcarbazepine. High RSD values suggest changes in consumption over time, either weekly, seasonal, or yearly trends.

### 3.2. Health- and Lifestyle-Related Statistics

**Figure 2:**
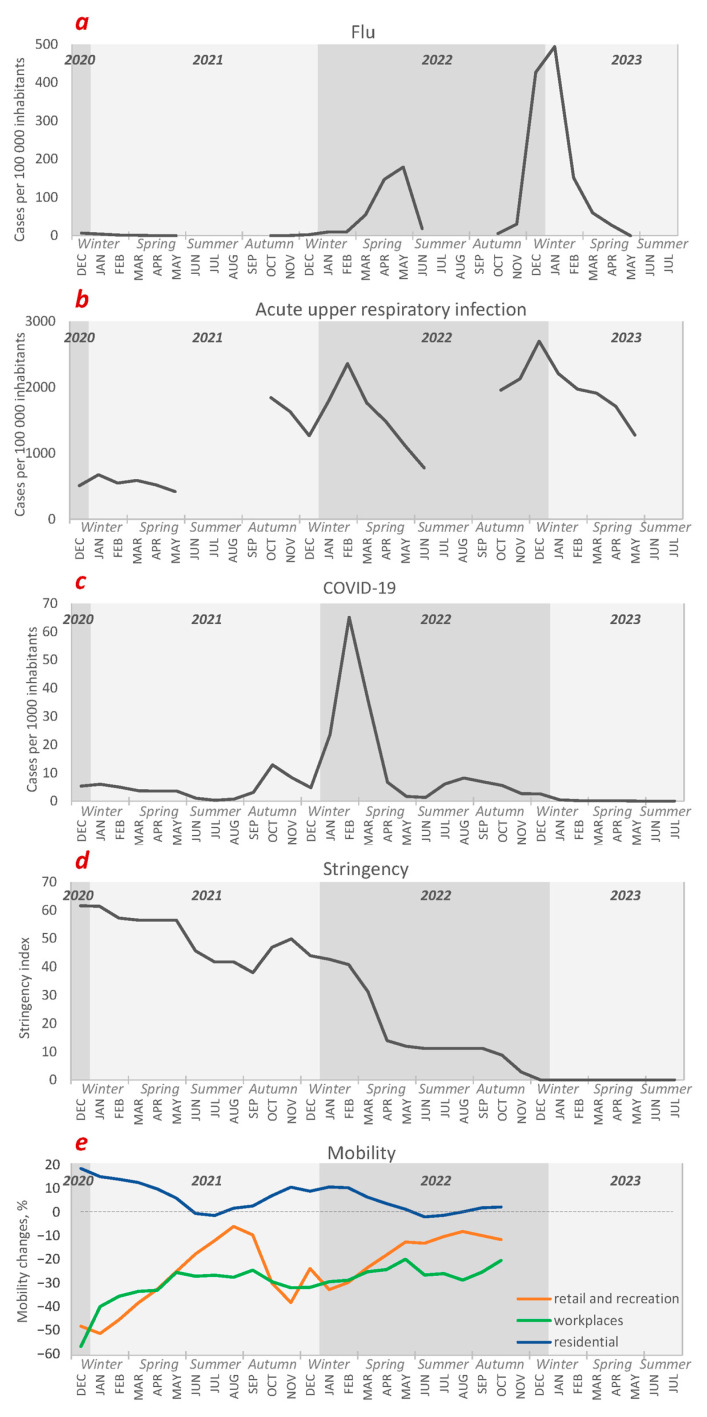
Health- and lifestyle-related statistics covering the period of the research, including (**a**) flu cases, (**b**) other acute upper respiratory infection cases, (**c**) COVID-19 cases, (**d**) stringency index, and (**e**) mobility changes relative to baseline.

In order to understand consumption patterns, relevant statistics of the reviewed period must be described. Since the analytes included in this study comprise various pharmaceuticals and recreational drugs, both health- and lifestyle-related statistics are of interest. Statistics on the flu, other acute upper respiratory infections, and confirmed COVID-19 cases could be useful for the interpretation of pharmaceutical consumption trends. Health-related statistics were gathered from the official website of the Centre for Disease Prevention and Control of Latvia (https://www.spkc.gov.lv/lv, accessed on 2 August 2023). Data on flu and acute upper respiratory infection cases were available only for the typical flu season periods—from October to May of each year. To simplify the reporting process, we summarized all the statistics that are relevant for data interpretation in Figure 2. As can be seen in Figure 2a,b, flu and other respiratory infection cases were significantly lower in 2021. Restrictions implemented to reduce COVID-19 risk and lower illness incidents, as well as difficulty to visit the hospital, may have led to incorrect statistical data during that period. On the contrary, in the 2022/2023 flu season, when the restrictions were eased, the number of flu cases more than doubled compared to the previous year.

The first COVID-19 wave in Latvia came in the autumn of 2020 and lasted until the beginning of the summer of 2021, reaching a peak morbidity of 7 cases per 1000 inhabitants in January. As can be seen in Figure 2c, there was a second wave during autumn of 2021 that reached a maximum of 17 cases per 1000 inhabitants at the end of October. The third wave of COVID-19 (Omicron variant) was the highest, reaching a peak morbidity of 68 cases per 1000 inhabitants in February of 2022.

In order to fight the pandemic, the Latvian government implemented various restrictions, such as mandatory masks, school closures, a public-gathering ban, lockdowns, and curfews. A useful indicator of the extent of government interventions to contain virus spread is the stringency index. It is a composite measure based on nine response indicators, including school closures, workplace closures, and travel bans, rescaled to a value from 0 to 100 (100 = strictest). Data were obtained from ourworldindata.org. As shown in Figure 2d, most restrictions were implemented at the beginning of the sampling campaign. In the summer of 2021, COVID-19 restrictions were relaxed. However, during the autumn of the same year, when the second COVID-19 wave hit, the restrictions were slightly increased. Despite the peak of the Omicron variant, the restrictions were not tightened any further. This was because over half of the population had been vaccinated, which led to the relaxation of restrictions. Significant alleviation occurred in April 2022, and all restrictions were cancelled by the end of 2022. Another indicator of the impact of governmental interventions on social life and behaviour is community mobility reports (Figure 2e). Data for the population of Riga were collected and provided by Google LLC (Mountain View, CA, USA, https://www.google.com/covid19/mobility/, accessed on 14 July 2023). The available data summarize the percent changes in visits to either places of residence, workplaces, or places of retail and recreation between December 2020 and October 2022 (reports were no longer updated). As can be observed, visits to retail and recreational places as well as workplaces were significantly lower in the winter of 2020 compared to the baseline. That corresponds to the previously described stringency index value, which was the highest in the same period. Similar negative correlations can be observed throughout the whole period. We hypothesized that changes in mobility and the imposed restrictions, in addition to COVID-19 morbidity, might have impacted pharmaceutical and recreational drug consumption trends, and WBE could be used to estimate these changes.

### 3.3. Consumption Patterns of Recreational Drugs

**Figure 3:**
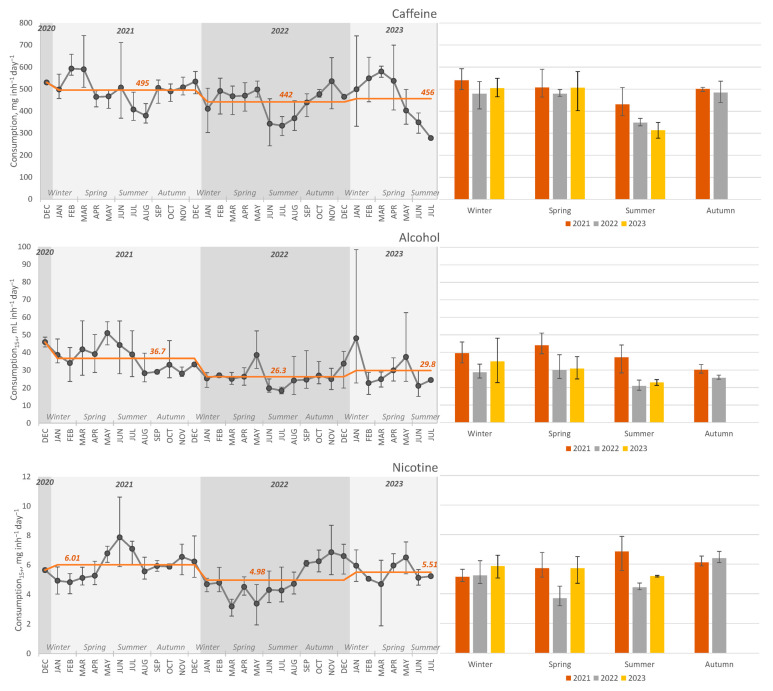
Consumption plots of recreational drugs during the sampling period as line charts (data points represent the monthly average, error bars represent monthly range, the orange line represents yearly average) and the seasonal average of each year as bar charts (error bars represent a range of monthly averages).

Caffeine, alcohol, and nicotine are among the most popular and widely consumed psychoactive or recreational drugs. Consumption graphs of three recreational drugs are shown in Figure 3.

#### 3.3.1. Caffeine

Caffeine consumption estimation was performed by measuring the concentration of the parent compound in raw wastewater, performing population normalisation, and applying an excretion factor. Average caffeine consumption was 468 mg inh^−1^ day^−1^ throughout the sampling period. In a recent study conducted in Innsbruck, Austria, values of 519 and 570 mg inh^−1^ day^−1^ were observed before and during the lockdown, respectively. The sample collection process was conducted daily for a period of up to one month. This allowed for a more precise estimation of average consumption, accounting for weekly trends [3]. The obtained values in both studies are higher than the recommended daily intake of 400 mg [26]. The reason behind the observed overestimation of caffeine consumption might be the use of the parent compound as a biomarker. Considering that caffeine can be present in wastewater from sources other than human metabolism, such as unconsumed coffee or coffee grounds, applying the excretion factor to the measured concentration data leads to a significant overestimation of overall consumption. This shortcoming could be avoided by using caffeine metabolites such as paraxanthine or 1,7-dimethyluric acid as biomarkers of caffeine consumption [27].

Seasonal trends of caffeine consumption are depicted in Figure 3. The average caffeine consumption is similar throughout the three sampling years. Significantly lower (*p*-value < 0.05) caffeine consumption was observed during the summer months compared to the rest of the period. It is consistent with the fact that most caffeine consumption occurs through coffee and tea, which are primarily hot beverages that are consumed more in colder weather [28]. An interesting finding was made by [29] in their research on caffeine consumption in a public university campus, with conclusions that monthly comparisons showed elevated levels of caffeine consumption in October, as well as for the first and last months of the academic year. We can assume that caffeine consumption can be impacted by students’ habits during the academic year and lower caffeine consumption during summer vacation. In the college student population, there is concern about overuse (>400 mg caffeine per day), mainly because of the increased popularity of energy drinks.

Moderate negative correlation was discovered between caffeine consumption and retail and recreational place mobility level (*r* = −0.63, *p*-value < 0.001), as well as moderate positive correlation with residential area mobility level—*r* = −0.67, *p* value < 0.001 (Table 1). This finding might indicate that increased caffeine consumption during pandemics occurred at home. Other authors have observed opposite correlation patterns in a small-sized community in Brazil [16]. Nevertheless, coffee consumption varies even among European Union countries due to shifts in cultural preferences, economic factors, and global trends [30,31].

#### 3.3.2. Alcohol

Alcohol consumption was estimated by measuring ethyl sulphate as a biomarker. It is the metabolite of ethanol that is stable and detectable in wastewater [32]. The average consumption throughout the whole sampling period was 31.6 mL inh_15+_^−1^ day^−1^ or 11.5 L inh_15+_^−1^ year^−1^. The observed value is slightly lower than the reported statistics or registered alcohol consumption in Latvia in 2021 and 2022 of 12.3 and 12.8 L inh_15+_^−1^ year^−1^, respectively [33]. Since the statistics are based on sales data, they do not account for the amount of alcohol purchased in Latvia but consumed elsewhere. Alcohol prices in Latvia are lower than in Estonia and Finland; therefore, there are many people who come to Latvia specifically to purchase alcoholic beverages. This assumption is supported by the fact that in 2021, when travel restrictions were in place, the average consumption calculated by WBE was higher than in 2022 (Figure 3), whereas the data from the sales statistics show the opposite trend. This might suggest that the alcohol consumption estimation by WBE is more accurate than the sales statistics.

As can be seen in Figure 3, there are two significantly elevated consumption values in January and May of 2023. The highest consumption was calculated on 3 January—98.4 mL inh_15+_^−1^ day^−1^; this was followed by 2 May—62.6 mL inh_15+_^−1^ day^−1^. Studies suggest that alcohol consumption tends to increase during holidays and festive occasions. This trend has been observed by several researchers who have found that alcohol consumption rises significantly during public holidays from December to January, as well as on national public holidays [34,35].

Lower alcohol consumption was observed during the summer months of 2022 and 2023. Other studies share contrary findings. Alcohol consumption was reported to be the highest during the summer months as well as during the holiday season during December and January [36,37]. Similar to caffeine consumption, this observation might point out the differences in alcohol consumption habits between different countries. Higher consumption during colder months might be due to increased drinking of liquors and spirits, whereas during summer, light alcoholic beverages such as beer are preferred. Furthermore, a lack of activities and events during winter might increase alcohol consumption at home or bars.

The elevated alcohol consumption during 2021 compared to 2022 and 2023 coincided with the pandemic and the implementation of the most severe restrictions. According to the correlation analysis, alcohol consumption correlates strongly with the stringency index (*r* = 0.70, *p*-value < 0.001), which is a measure of the extent of restrictions (Table 1). That indicates that the restrictions might have caused an increase in alcohol consumption. Other authors have observed an opposite relationship—due to the shutdown of the hospitality industry and cancellation of events, common places and occasions for social drinking of alcoholic beverages were not available; consequently, alcohol consumption in Innsbruck, Austria, dropped from 12.6 to 9.9 kg day^−1^ 1000 inh^−1^ [3], and in selected Slovak cities during the lockdown, it was reported to decrease ca 20% compared to the period of milder restrictions [38]. However, it is important to note that the relationship between pandemic limitations and alcohol consumption is complex. As demonstrated by the example of Innsbruck, Austria, some districts experienced a decline in alcohol consumption as a result of the closure of popular sites for social drinking. This highlights how the impact of alcohol consumption limitations varies depending on local settings and cultural norms. The same conclusion was reported by Krisjane et al. [39], that the data on studies regarding the crucial challenges and immediate impact of the COVID-19 pandemic on mental health and the quality of life of individuals and families worldwide are incomplete, sometimes controversial, and may have a significant cultural influence. According to the data obtained in our study regarding the Latvian population, harsh restrictions contributed to an increase in alcohol use. The limitations may have exacerbated worry and psychological anguish, forcing some people to drink more alcohol to cope with their feelings.

#### 3.3.3. Nicotine

Cotinine served as a biomarker for estimating nicotine consumption. The average nicotine consumption throughout the whole sampling period was 5.50 mg inh_15+_^−1^ day^−1^, which corresponds to 4.4 cig inh_15+_^−1^ day^−1^ (1.25 mg nicotine per cigarette [3,40]). For comparison purposes, nicotine consumption expressed for the whole population was calculated as well—4.62 mg inh^−1^ day^−1^. Accurate nicotine consumption data for Latvia are unavailable. However, taking into account the percentage of daily smokers among the population of those 15 years and older in Latvia in 2019 (22.8% [41]) and the average daily intake of nicotine per smoker (25 mg day^−1^ [42]), it can be calculated that the average nicotine consumption in Latvia is 5.70 mg inh_15+_^−1^ day^−1^. As a result, it can be concluded that the nicotine consumption estimated by the WBE approach is in reasonable agreement with the statistics.

As can be seen in Figure 3, the yearly average consumption trends were similar to those of alcohol—higher nicotine consumption occurred during 2021, when most restrictions were in place. The highest values were obtained in the summer of 2021, when several pandemic restrictions were eased, suggesting an increase in social smoking. Lower nicotine consumption was observed during the spring and summer of 2022, which might suggest that restriction cancellation reduced stress-related smoking. An increase in nicotine consumption was observed in the spring of 2023; however, further research is necessary to fully understand the reasons behind it.

### 3.4. Consumption Patterns of Pharmaceuticals

**Figure 4:**
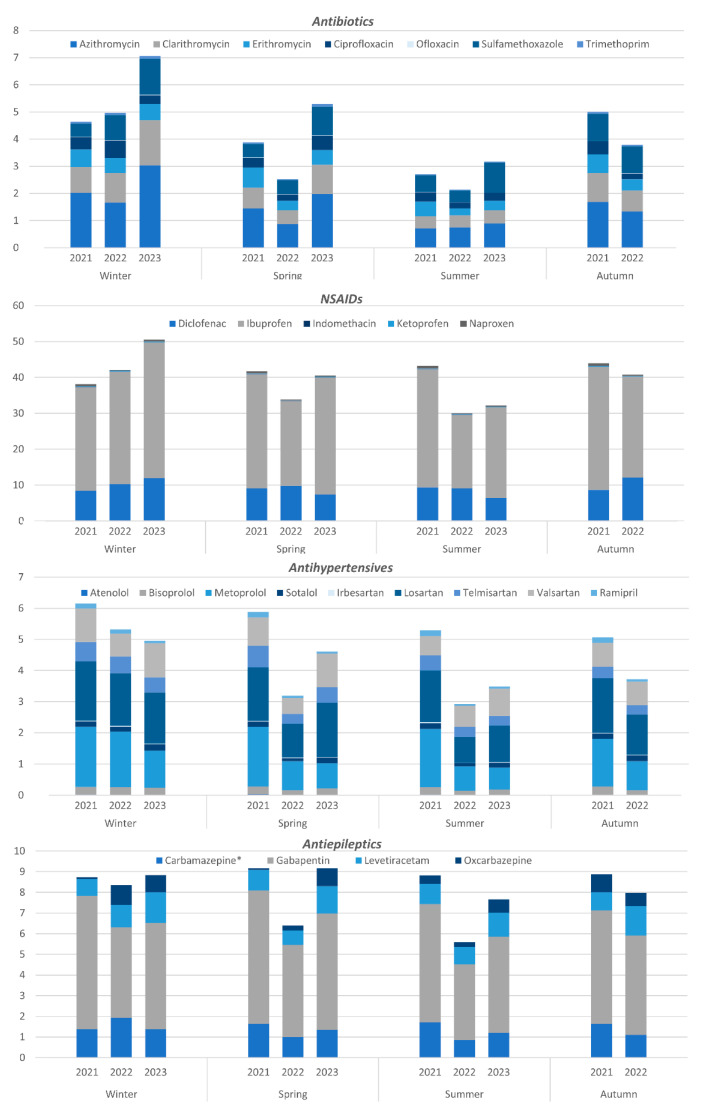
Average seasonal consumption in mg inh^−1^ day^−1^ of four main groups of pharmaceuticals included in the study (* for carbamazepine the average value calculated from the content of the parent compound and carbamazepine epoxide as a biomarker is shown).

A graphical depiction of seasonal variations in monthly consumption among four main drug classes included in the study is shown in Figure 4. All individual graphs are included in the Supporting Information file, representing seasonal variations of selected compounds, the yearly average consumption estimated by WBE, and consumption data from sales statistics. In some cases, the average consumption estimated by the two methods significantly differs. Experimental uncertainties accompany every method, leading to potential over- or underestimation of true values. There are several reasons why drug estimates may be inaccurate. These reasons include the direct disposal of unused drugs into sewage systems, underestimated or overestimated analyte excretion by humans, uncertainties related to analyte biotransformation, the recovery rate of analytical methods, or analyte losses [43].

#### 3.4.1. Antibiotics

Three types of antibacterial agents were included in the study—macrolides, fluoroquinolones, and sulphonamides. The highest consumption rates were observed for the macrolide antibiotics azithromycin and clarithromycin—on average, 1.51 and 0.85 mg inh^−1^ day^−1^ or 3.01 and 1.71 DDD 1000 inh^−1^ day^−1^, respectively. This is consistent with the drug consumption statistics data, where, among the three antibiotic groups included in this study, macrolide consumption is the highest [44].

A strong temporal trend of lower antibiotic use during summer was observed, as expected, due to a higher incidence of respiratory illnesses during winter months. Similar observations have been made by other authors [43,45,46]. A strong positive correlation was observed between the use of sulfamethoxazole and acute upper respiratory infection cases—*r* = 0.84, *p*-value < 0.001 (Table 2). For other antibiotics, such as clarithromycin, only moderate correlations were discovered. Sulfamethoxazole is used for the treatment of bacterial infections such as urinary tract infections and bronchitis, which are both seasonal infections with peaks in winter. Another explanation regarding strong correlations is that bronchitis has a sudden onset and usually appears after a respiratory infection, such as a cold or flu-like illness. In the case of clarithromycin, it is the most prescribed antibiotic for the treatment of respiratory tract infections in combination with amoxicillin [47]. The analysis of the consumption of antibiotics in the EU community revealed the seasonality trend as well. While a limited amount of seasonal variation could be associated with seasonality in bacterial pathogens, the extent of the observed seasonality suggests inappropriate usage for viral (mostly respiratory) infections during the winter season [48].

Other authors observed an increased use of azithromycin during the pandemic [15]. In our study, only a moderate correlation between COVID-19 cases and antibiotic consumption was observed, and no correlation with azithromycin was found. This might suggest differences in treatment approaches between different countries.

Interesting findings were discovered in regard to the correlation between antibiotic consumption and mobility reports. Moderate and strong positive correlations were observed with mobility changes in residential area visits, which, in other words, means visiting close friends and relatives during the pandemic (*r* = 0.59–0.85, *p*-value < 0.05). Contrarily, negative correlations were observed with changes in retail and recreational place visits (*r* = −0.57–−0.82, *p*-value < 0.05).

#### 3.4.2. Non-Steroidal Anti-Inflammatory Drugs

Among the analysed NSAIDs, a strong dominance in the consumption of ibuprofen and diclofenac was observed—on average, 29.9 and 9.40 mg inh^−1^ day^−1^ or 24.9 and 94.0 DDD 1000 inh^−1^ day^−1^, respectively. This observation is in reasonable agreement with statistics, where ibuprofen and diclofenac contribute the most to the total consumption of NSAIDs. Moreover, the consumption of ibuprofen determined by WBE is very close to the statistics—25.8 DDD 1000 inh^−1^ day^−1^ (average consumption during 2020–2022). In the case of diclofenac, the consumption value determined by the WBE approach is significantly higher than the statistics—17.2 DDD 1000 inh^−1^ day^−1^ [44]. Since diclofenac is widely used in the form of a topical gel, it might enter the sewage system in higher amounts than predicted by excretion rates; therefore, the WBE approach results in elevated consumption levels.

No distinct seasonal patterns of the consumption of NSAIDs were observed (Figure 4). However, by looking at the individual consumption graphs (Supporting Information), it is evident that apart from 2021, ibuprofen consumption is lower during the summer, indicating that the pandemic presumably influenced the typical consumption pattern. This assumption was further supported by correlation analysis, which revealed a moderate positive correlation between ibuprofen consumption and stringency index.

#### 3.4.3. Antihypertensives

Three types of antihypertensive agents were included in this study—ACE inhibitors, angiotensin II receptor blockers, and beta-blockers. The most consumed antihypertensives were the angiotensin II receptor blocker losartan and beta-blocker metoprolol—1.51 and 1.33 mg inh^−1^ day^−1^ or 30.1 and 8.84 DDD 1000 inh^−1^ day^−1^, respectively. However, taking into account the DDD of each compound, ACE inhibitor ramipril consumption was the highest—46.3 DDD 1000 inh^−1^ day^−1^. This observation is confirmed by sales statistics, where ramipril has the highest consumption among the analysed compounds—26.4 DDD 1000 inh^−1^ day^−1^ [44]. However, the value determined by WBE is almost two-times higher than reported by statistics. The overestimation could be caused by several reasons. First, as described before, high uncertainty of consumption estimation by WBE is introduced by the excretion factor—the number of studies regarding human pharmacokinetics is very limited [20]. Second, sales statistics are calculated only for individual substances and not for combinations. Therefore, the statistics reflect only approximately 60% of overall drug use and could lead to an underestimation of actual consumption.

The seasonal variation in antihypertensive use is displayed in Figure 4. Apart from 2021, lower consumption was observed during warm seasons. It is consistent with the well-known relationship between environmental temperatures and blood pressure—lower blood pressure levels are associated with higher environmental temperatures, and vice versa [49]. Higher consumption was recorded in 2021 compared to 2022 and 2023—164 mg inh^−1^ day^−1^ versus 97 and 115 mg inh^−1^ day^−1^, respectively. The most pronounced differences were detected during spring and summer, where no significant difference between the seasons in 2021 was found. Similar trends were observed regarding the previously described consumption of NSAIDs. Moreover, strong positive correlations were found between most antihypertensives and the stringency index (Table 2), indicating that the implemented restrictions could have caused increased hypertension incidence. This observation could be explained by increased stress during the pandemic conditions, since stress has been known to raise blood pressure and lead to hypertension [50].

#### 3.4.4. Antiepileptics

The most consumed pharmaceutical among the four analysed antiepileptics was gabapentin—5.18 mg inh^−1^ day^−1^ or 2.88 DDD 1000 inh^−1^ day^−1^. This is in good agreement with the sales data, where gabapentin has the highest consumption rate among the analysed compounds—3.41 DDD 1000 inh^−1^ day^−1^ [44].

The overall consumption of antiepileptics throughout the year was steady, except for the spring and summer of 2022, when a decrease was observed. Due to the absence of reference statistics regarding the seasonal consumption of antiepileptics, no conclusions on the applicability of WBE for consumption estimation can be drawn. Other authors have observed similar levels of carbamazepine throughout the year, which is consistent with our findings, except for 2022 [45].

Very distinctive trends were observed for oxcarbazepine. Significant increases in use were observed from June 2021 to March 2022 and again from August 2022 to June 2023 (see Supporting Information). Interestingly, no such trend was found for any other antiepileptic.

The correlation analysis revealed a moderate positive correlation between carbamazepine use and stringency index—*r* = 0.65, *p*-value < 0.001. Other authors also observed an increased use of carbamazepine during the lockdown compared to the pre-pandemic period [15]. However, no significant influence of COVID-19 was observed on the consumption of antiepileptics.

#### 3.4.5. Other Pharmaceuticals

Consumption graphs for all other pharmaceuticals are available in the Supporting Information file.

The most pronounced seasonal trends were observed in the case of the antiasthmatic drug salbutamol and the decongestant xylometazoline, both peaking during the colder seasons of autumn, winter, and spring, as expected. A notable decrease in consumption during summer was also observed for antidiabetic metformin. Although metformin consumption is expected to be constant throughout the year, some findings show that the drug’s efficiency is lower if vitamin D levels in the blood are low [51]. Therefore, it is possible that higher doses are necessary during periods of low sunlight. In another study, the WBE approach was applied to estimate the consumption of metformin in Dalina over a four-year period. The report indicates significant differences in the concentrations of metformin in water during different seasons, with 94.7 μg/L in spring, 18.1 μg/L in summer, 47.8 μg/L in autumn, and 79.0 μg/L in winter. However, the back-calculated data for daily consumption showed no significant difference between seasons. The study estimated the population size based on the ammonium content found in wastewater [52].

No seasonality was observed in the case of the analgesic acetaminophen, antiviral acyclovir, and antihyperlipidemics atorvastatin and rosuvastatin, indicating constant use throughout the year. As regards the impact of COVID-19, only atorvastatin demonstrated a strong positive correlation with the stringency index. However, it is most probably due to other reasons that caused a decrease in atorvastatin use in 2022.

The most significant impact of COVID-19 and the implemented restrictions was observed in the case of the opioid tramadol, psychiatric amisulpride, and antidepressant venlafaxine. Similar observations were made by Montgomery et al. [53], indicating that the restrictions might have resulted in a higher incidence of mental health problems. These observations are confirmed by a comprehensive review of 3166 papers on the psychological impact of quarantine, indicating reported negative psychological effects, including confusion, anger, and different stressors, such as longer quarantine duration, infection fears, frustration, boredom, inadequate supplies, inadequate information, financial loss, and stigma [54].

## 4. Conclusions

This study aimed to identify trends in the usage patterns of pharmaceutics and recreational drugs during the COVID-19 lockdown in Riga, the capital of Latvia, by using the WBE approach. In total, 40 biomarkers were selected for the present study and quantified in wastewater samples over a period of 32 months using the heart-cutting 2D-HPLC-MS/MS method. The consumption trends were estimated by back-calculation from measured biomarker concentrations in wastewater samples.

All 40 targeted compounds were detected in each composite wastewater sample. The obtained back-calculated consumption ranges spanned 6 orders of magnitude. Caffeine was the most abundant compound analysed; an average value of 470,671 mg 1000 inh^−1^ day^−1^ was obtained, followed by the antidiabetic drug metformin, 30,414 mg 1000 inh^−1^ day^−1^, the pain medication ibuprofen, 29,731 mg 1000 inh^−1^ day^−1^, and alcohol, 21,100 mg 1000 inh^−1^ day^−1^. The lowest consumption rates were observed in the case of the antiasthmatic drug salbutamol, 5.25 mg 1000 inh^−1^ day^−1^, and the decongestant xylometazoline, 7.06 mg 1000 inh^−1^ day^−1^.

The impact of the COVID-19 pandemic and the consequential restrictions on consumption trends was evaluated using correlation analysis. Among recreational drugs, the influence of the pandemic restrictions was observed on the consumption of all three substances—alcohol, nicotine, and caffeine. For caffeine and alcohol consumption, positive correlations were observed with the level of restrictions. Although nicotine did not demonstrate any correlations, the consumption trends were similar to those of alcohol. It can be concluded that the drinking and smoking habits of the population were negatively impacted by restrictions, which suggests a complex interplay between stress, coping strategies, changing social dynamics, and leisure activities.

The research also revealed changes in medication use trends. During the pandemic, there was a rise in the intake of specific pharmaceuticals, including antihypertensives, antidepressants, psychiatrics, and ibuprofen, particularly while strict restrictions were in place. This suggests that the epidemic and restrictions may have had an effect on the population’s health and mental well-being, resulting in changes in medicine consumption. The findings of this study align with a report on the mental health of Latvian adolescents and young people during the COVID-19 pandemic. The report confirmed that pandemic restrictions had a serious impact on mental health. The obtained data showed that more than 40% of respondents experienced learning difficulties and depression, faced obsessive thoughts, suffered from irritability, and had an increased feeling of loneliness [55,56].

Distinct seasonal trends were discovered in the consumption patterns of antibiotics, the antiasthmatic drug salbutamol, and the decongestant xylometazoline, where higher consumption occurred during colder seasons, as expected. Interestingly, apart from 2021, non-steroidal anti-inflammatory drugs and antihypertensives were also used less during the summer months. The different consumption patterns during 2021, when the restriction level was the highest, suggest that the pandemic affected the consumption patterns of these pharmaceuticals as well.

The present study elucidates the multidimensional impact of the COVID-19 epidemic and accompanying limitations on recreational and medication drug consumption patterns. In addition to revealing a new perspective on public health behaviour, WBE provides an opportunity to investigate the complicated relationship between changing circumstances within a community, stress experienced during a pandemic, and some sociocultural differences between countries.

## Figures and Tables

**Table 1:** Correlation coefficients (*r*) obtained by simple correlation analysis between daily consumption of recreational drugs and lifestyle-related statistics (only significant correlations are shown (*p*-value < 0.05)).

Compound	Stringency	Mobility		
		Retail and Recreation	Workplaces	Residential
Alcohol ^a^	0.70	−0.44	−0.40	0.39
Caffeine	0.46	−0.63	−0.37	0.67
Nicotine ^b^				

^a^ determined as ethyl sulphate, ^b^ determined as cotinine.

**Table 2:** Correlation coefficients (*r*) obtained by simple correlation analysis between daily pharmaceutical consumption and health and lifestyle related statistics (only significant correlations are shown (*p*-value < 0.05)).

Compound	Therapeutic Group	Flu	Acute upper Respiratory Infection	COVID-19	Stringency	Mobility		
						Retail and Recreation	Workplaces	Residential
Ciprofloxacin	Fluoroquinolone antibiotic	−0.38		0.56	0.63	−0.59	−0.46	0.61
Ofloxacin					0.36	−0.57	−0.58	0.59
Azithromycin	Macrolide antibiotic	0.54	0.43		0.54	−0.82	−0.60	0.85
Clarithromycin		0.56	0.55	0.40	0.46	−0.67	−0.36	0.71
Erythromycin					0.74	−0.65	−0.49	0.64
Sulfamethoxazole	Sulphanilamide antibiotic	0.57	0.84					
Trimethoprim			0.49	0.55	0.49	−0.60	−0.45	0.69
Gabapentin	Antiepileptic		−0.43		0.57	−0.41	−0.45	0.35
Carbamazepine ^a^				0.41	0.65			0.37
Levetiracetam		0.35	0.58					
Oxcarbazepine			0.73	0.37				
Atorvastatin	Antihyperlipidemic		−0.42		0.81	−0.68	−0.65	0.64
Rosuvastatin		0.53	0.49	0.46		−0.42		0.38
Ramipril	ACE inhibitor	−0.36	−0.52		0.81	−0.42	−0.39	0.37
Irbesartan	Angiotensin blocker	−0.40	−0.44		0.81	−0.52	−0.42	0.46
Losartan					0.82	−0.66	−0.51	0.64
Telmisartan			−0.43		0.78	−0.68	−0.58	0.62
Valsartan					0.53	−0.74	−0.67	0.70
Atenolol	Beta-blocker		−0.69	−0.36	0.74	−0.54	−0.51	0.44
Bisoprolol					0.81	−0.56	−0.49	0.54
Metoprolol			−0.40		0.88	−0.59	−0.59	0.57
Sotalol					0.40			
Diclofenac	NSAID	0.46	0.48		−0.35			
Ibuprofen		0.40	0.39		0.60			
Indomethacin						−0.40	−0.38	0.36
Ketoprofen				0.41	0.53	−0.49	−0.37	0.49
Naproxen					0.47			
Acetaminophen	Analgesic			0.56	0.42			0.40
Salbutamol	Antiasthmatic		0.59					
Venlafaxine ^b^	Antidepressant				0.68	−0.36		
Metformin	Antidiabetic				0.56	−0.57		0.56
Acyclovir	Antiviral							
Xylometazoline	Decongestant			0.42				0.41
Tramadol	Opioid				0.81	−0.48	−0.43	0.46
Amisulpride	Psychiatric				0.74	−0.38		0.35

^a^ average consumption calculated from carbamazepine and carbamazepine-10,11-epoxide, ^b^ average consumption calculated from venlafaxine and O-desmethylvenlafaxine.

## Data Availability

Data are contained within the article and Supplementary Materials.

## Supplementary Materials

The following supporting information can be downloaded at: https://www.mdpi.com/article/10.3390/ijerph21020206/s1, Figure S1: Seasonal variations of antibiotic consumption (DDD 1000 inh^−1^ day^−1^): (a) macrolides; (b) fluoroquinolones, and (c) sulphanilamides; Figure S2: Seasonal variations of NSAID consumption; Figure S3: Seasonal variations of antiepileptic consumption; Figure S4: Seasonal variations of antiepileptic consumption (DDD 1000 inh^−1^ day^−1^): (a) ACE inhibitor; (b) angiotensin II receptor blockers, and (c) beta-blockers; Figures S5 and S6: Seasonal variations of the consumption of other pharmaceuticals included in the study; Table S1: Parameters used for consumption calculation; Table S2: Concentrations and corresponding consumptions of the detected compounds in tested wastewater samples. References [57,58,59,60,61,62,63,64,65,66,67,68,69,70,71,72,73,74,75,76,77,78,79,80,81,82,83,84,85,86,87,88,89,90,91,92,93,94,95,96,97] are cited in supplementary material.

## References

[1] Alygizakis N., Galani A., Rousis N.I., Aalizadeh R., Dimopoulos M.A., Thomaidis N.S. (2021). Change in the chemical content of untreated wastewater of Athens, Greece under COVID-19 pandemic. Sci. Total Environ..

[2] Picó Y., Barceló D. (2023). Microplastics and other emerging contaminants in the environment after COVID-19 pandemic: The need of global reconnaissance studies. Curr. Opin. Environ. Sci. Health.

[3] Reinstadler V., Ausweger V., Grabher A.L., Kreidl M., Huber S., Grander J., Haslacher S., Singer K., Schlapp-Hackl M., Sorg M. (2021). Monitoring drug consumption in Innsbruck during coronavirus disease 2019 (COVID-19) lockdown by wastewater analysis. Sci. Total Environ..

[4] Rousis N.I., Gracia-Lor E., Hernández F., Poretti F., Santos M.M., Zuccato E., Castiglioni S. (2021). Wastewater-based epidemiology as a novel tool to evaluate human exposure to pesticides: Triazines and organophosphates as case studies. Sci. Total Environ..

[5] Gudra D., Dejus S., Bartkevics V., Roga A., Kalnina I., Strods M., Rayan A., Kokina K., Zajakina A., Dumpis U. (2022). Detection of SARS-CoV-2 RNA in wastewater and importance of population size assessment in smaller cities: An exploratory case study from two municipalities in Latvia. Sci. Total Environ..

[6] Jones H.E., Hickman M., Kasprzyk-Hordern B., Welton N.J., Baker D.R., Ades A.E. (2014). Illicit and pharmaceutical drug consumption estimated via wastewater analysis. Part B: Placing back-calculations in a formal statistical framework. Sci. Total Environ..

[7] Choi P.M., Tscharke B., Samanipour S., Hall W.D., Gartner C.E., Mueller J.F., Thomas K.V., O’Brien J.W. (2019). Social, demographic, and economic correlates of food and chemical consumption measured by wastewater-based epidemiology. Proc. Natl. Acad. Sci. USA.

[8] Zhang Y., Duan L., Wang B., Du Y., Cagnetta G., Huang J., Blaney L., Yu G. (2019). Wastewater-based epidemiology in Beijing, China: Prevalence of antibiotic use in flu season and association of pharmaceuticals and personal care products with socioeconomic characteristics. Environ. Int..

[9] Perkons I., Tomsone L.E., Sukajeva V., Neilands R., Kokina K., Pugajeva I. (2022). Qualitative fingerprinting of psychoactive pharmaceuticals, illicit drugs, and related human metabolites in wastewater: A year-long study from Riga, Latvia. J. Environ. Chem. Eng..

[10] Boogaerts T., Ahmed F., Choi P.M., Tscharke B., O’Brien J., De Loof H., Gao J., Thai P., Thomas K., Mueller K. (2021). Current and future perspectives for wastewater-based epidemiology as a monitoring tool for pharmaceutical use. Sci. Total Environ..

[11] Mishra N.N.P., Das S.S., Yadav S., Khan W., Afzal M., Alarifi A., Kenawy E.R., Ansari M.T., Hasnain M.S., Nayak A.K. (2020). Global impacts of pre- and post-COVID-19 pandemic: Focus on socio-economic consequences. Sens. Int..

[12] Sokolović D., Drakul D., Vujić-Aleksić V., Joksimović B., Marić S., Nežić L. (2023). Antibiotic consumption and antimicrobial resistance in the SARS-CoV-2 pandemic: A single-center experience. Front. Pharmacol..

[13] Morales-Paredes C.A., Rodríguez-Díaz J.M., Boluda-Botella N. (2022). Pharmaceutical compounds used in the COVID-19 pandemic: A review of their presence in water and treatment techniques for their elimination. Sci. Total Environ..

[14] Jiménez-Bambague E.M., Madera-Parra C.A., Machuca-Martinez F. (2023). The occurrence of emerging compounds in real urban wastewater before and after the COVID-19 pandemic in Cali, Colombia. Curr. Opin. Environ. Sci. Health.

[15] Galani A., Alygizakis N., Aalizadeh R., Kastritis E., Dimopoulos M.A., Thomaidis N.S. (2021). Patterns of pharmaceuticals use during the first wave of COVID-19 pandemic in Athens, Greece as revealed by wastewater-based epidemiology. Sci. Total Environ..

[16] Hahn R.Z., Bastiani M.F., Lizot L.L.F., Schneider A., da Silva Moreira I.C., Meireles Y.F., Viana M.F., do Nascimento C.A., Linden R. (2022). Long-term monitoring of drug consumption patterns during the COVID-19 pandemic in a small-sized community in Brazil through wastewater-based epidemiology. Chemosphere.

[17] Pelham W.E., Yuksel D., Tapert S.F., Baker F.C., Pohl K.M., Thompson W.K., Podhajsky S., Reuter C., Zhao Q., Eberson-Shumate S.C. (2022). Did the acute impact of the COVID-19 pandemic on drinking or nicotine use persist? Evidence from a cohort of emerging adults followed for up to nine years. Addict. Behav..

[18] Pazzagli L., Reutfors J., Lucian E., Zerial G., Perulli A., Castelpietra G. (2022). Increased antidepressant use during the COVID-19 pandemic: Findings from the Friuli Venezia Giulia region, Italy, 2015–2020. Psychiatry Res..

[19] Yavuz-Guzel E., Atasoy A., Gören İ.E., Daglioglu N. (2022). Impact of COVID-19 pandemic on antidepressants consumptions by wastewater analysis in Turkey. Sci. Total Environ..

[20] Tomsone L.E., Perkons I., Sukajeva V., Neilands R., Kokina K., Bartkevics V., Pugajeva I. (2022). Consumption trends of pharmaceuticals and psychoactive drugs in Latvia determined by the analysis of wastewater. Water Res..

[21] Chen C., Kostakis C., Gerber J.P., Tscharke B.J., Irvine R.J., White J.M. (2014). Towards finding a population biomarker for wastewater epidemiology studies. Sci. Total Environ..

[22] Thai P.K., O’Brien J.K., Banks A.P.W., Jiang G., Gao J., Choi P.M., Yuan Z., Mueller J.F. (2019). Evaluating the in-sewer stability of three potential population biomarkers for application in wastewater-based epidemiology. Sci. Total Environ..

[23] Pugajeva I., Ikkere L.E., Jansons M., Perkons I., Sukajeva V., Bartkevics V. (2021). Two-dimensional liquid chromatography—Mass spectrometry as an effective tool for assessing a wide range of pharmaceuticals and biomarkers in wastewater-based epidemiology studies. J. Pharm. Biomed. Anal..

[24] Gracia-Lor E., Castiglioni S., Bade R., Been F., Castrignanò E., Covaci A., González-Mariño I., Hapeshi E., Kasprzyk-Hordern B., Kinyua J. (2017). Measuring biomarkers in wastewater as a new source of epidemiological information: Current state and future perspectives. Environ. Int..

[25] Eurostat (2023). Population by Age Group. https://ec.europa.eu/eurostat/databrowser/view/TPS00010/default/table?lang=en.

[26] EFSA Panel on Dietetic Products, Nutrition and Allergies (NDA) (2015). Scientific Opinion on the safety of caffeine. EFSA J..

[27] Rousis N., Bade R., Gracia-Lor E. (2023). Wastewater-based epidemiology as a surveillance tool to assess human consumption of psychotropic substances: Alcohol, nicotine and caffeine as case studies. TrAC Trends Anal. Chem..

[28] Szabo de Edelenyi F., Druesne-Pecollo N., Arnault N., González R., Buscail C., Galan P. (2016). Characteristics of Beverage Consumption Habits among a Large Sample of French Adults: Associations with Total Water and Energy Intakes. Nutrients.

[29] Driver E.M., Gushgari A., Chen J., Halden R.U. (2020). Alcohol, nicotine, and caffeine consumption on a public U.S. university campus determined by wastewater-based epidemiology. Sci. Total Environ..

[30] CBI, Ministry of Foreign Affairs (2022). What Is the Demand for Coffee on the European Market?. https://www.cbi.eu/market-information/coffee/what-demand#which-european-markets-offer-most-opportunities-for-coffee.

[31] DePaula J., Farah A. (2019). Caffeine Consumption through Coffee: Content in the Beverage, Metabolism, Health Benefits and Risks. Beverages.

[32] Reid M.J., Langford K.H., Mørland J., Thomas K.V. (2011). Analysis and interpretation of specific ethanol metabolites, ethyl sulfate, and ethyl glucuronide in sewage effluent for the quantitative measurement of regional alcohol consumption. Alcohol. Clin. Exp. Res..

[33] SPKC (2023). The Consumption of Registered Absolute Alcohol in Latvia [Reģistrētā Absolūtā Alkohola Patēriņš Latvijā]. https://www.spkc.gov.lv/lv/media/17567/download.

[34] Boogaerts T., Bertels X., Pussig B., Quireyns M., Toebosch L., Van Wichelen N., Dumitrascu C., Matheï C., Lahousse L., Aertgeerts B. (2022). Evaluating the impact of COVID-19 countermeasures on alcohol consumption through wastewater-based epidemiology: A case study in Belgium. Environ. Int..

[35] Zheng Q., Tscharke B.J., Krapp C., O’Brien J.W., Mackie R.S., Connor J., Mueller J.F., Thomas K.V., Thai P.K. (2020). New approach for the measurement of long-term alcohol consumption trends: Application of wastewater-based epidemiology in an Australian regional city. Drug Alcohol Depend..

[36] Olson K.L., Whitley P., Velasco J., LaRue L., Dawson E., Huskey A. (2021). Seasonal and Regional Influences on Alcohol Consumption: An Analysis of Near-Real-Time Urine Drug Test Results in Those Seeking Health Care. Drug Alcohol Depend..

[37] Knudsen A.K., Skogen J.C. (2015). Monthly variations in self-report of time-specified and typical alcohol use: The Nord-Trøndelag Health Study (HUNT3). BMC Public Health.

[38] Bimová P., Tulipánová A., Bodík I., Fehér M., Pavelka M., Castiglioni S., Zuccato E., Salgueiro-González N., Petrovičová N., Híveš J. (2023). Monitoring Alcohol Consumption in Slovak Cities during the COVID-19 Lockdown by Wastewater-Based Epidemiology. Int. J. Environ. Res. Public Health.

[39] Krisjane Z., Apsite-Berina E., Berzins M., Skadins T., Burgmanis G. (2020). Work-life balance during the COVID-19 outbreak: The case of Latvia. Balt. Reg..

[40] Prochaska J.J., Benowitz N.L. (2019). Current advances in research in treatment and recovery: Nicotine addiction. Sci. Adv..

[41] Eurostat (2022). Smoking of Tobacco Products by Sex, Age and Country of Citizenship. https://ec.europa.eu/eurostat/databrowser/view/HLTH_EHIS_SK1C__custom_7007340/default/table?lang=en&page=time:2019.

[42] Fagerström K. (2005). The nicotine market: An attempt to estimate the nicotine intake from various sources and the total nicotine consumption in some countries. Nicotine Tob. Res..

[43] Holton E., Louw C., Archer E., Louw T., Wolfaardt G., Kasprzyk-Hordern B. (2023). Quantifying community-wide antibiotic usage via urban water fingerprinting: Focus on contrasting resource settings in South Africa. Water Res..

[44] State Agency of Medicines (2023). Statistics on Medicines Consumption in Latvia from 2018 to 2022. [Zāļu Patēriņš pēc DDD uz 1000 Iedzīvotājiem Dienā 2018–2022. Gadā]. https://www.zva.gov.lv/lv/publikacijas-un-statistika/zalu-paterina-statistika-ddd.

[45] Golovko O., Kumar V., Fedorova G., Randak T., Grabic R. (2014). Seasonal changes in antibiotics, antidepressants/psychiatric drugs, antihistamines and lipid regulators in a wastewater treatment plant. Chemosphere.

[46] Im J.K., Kim S.H., Kim Y.S., Yu S.J. (2021). Spatio-Temporal Distribution and Influencing Factors of Human and Veterinary Pharmaceuticals in the Tributary Surface Waters of the Han River Watershed, South Korea. Int. J. Environ. Res. Public Health.

[47] Dumpis U., Dimiņa E., Akermanis M., Tirāns E., Veide S. (2013). Assessment of antibiotic prescribing in Latvian general practitioners. BMC Fam. Pract..

[48] Bruyndonckx R., Adriaenssens N., Versporten A., Hens N., Monnet D.L., Molenberghs G., Goossens H., Weist K., Coenen S., ESAC-Net Study Group (2021). Consumption of antibiotics in the community, European Union/European Economic Area, 1997–2017. J. Antimicrob. Chemother..

[49] Stergiou G.S., Palatini P., Modesti P.A., Asayama K., Asmar R., Bilo G., de la Sierra A., Dolan E., Head G., Kario K. (2020). Seasonal variation in blood pressure: Evidence, consensus and recommendations for clinical practice. Consensus statement by the European Society of Hypertension Working Group on Blood Pressure Monitoring and Cardiovascular Variability. J. Hypertens..

[50] Spruill T.M. (2010). Chronic psychosocial stress and hypertension. Curr. Hypertens. Rep..

[51] Krysiak R., Kowalcze K., Szkróbka W., Okopień B. (2023). Vitamin D Status Determines the Impact of Metformin on Gonadotropin Levels in Postmenopausal Women. J. Clin. Med..

[52] Xiao Y., Shao X.T., Tan D.Q., Yan J.H., Pei W., Wang Z., Yang M., Wang D.G. (2019). Assessing the trend of diabetes mellitus by analyzing metformin as a biomarker in wastewater. Sci. Total Environ..

[53] Montgomery A.B., Bowers I., Subedi B. (2021). Trends in Substance Use in Two United States Communities during Early COVID-19 Lockdowns Based on Wastewater Analysis. Environ. Sci. Technol. Lett..

[54] Brooks S.K., Webster R.K., Smith L.E., Woodland L., Wessely S., Greenberg N., Rubin G.J. (2020). The psychological impact of quarantine and how to reduce it: Rapid review of the evidence. Lancet.

[55] Institute of Economics of the Latvian Academy of Sciences (2021). Latvia Social Briefing, COVID-19: Mental Health of Latvian Adolescents and Young People. Weekly Briefing, Vol. 39, No. 3. https://china-cee.eu/2021/04/26/latvia-social-briefing-covid-19-mental-health-of-latvian-adolescents-and-young-people/.

[56] Konstantinovs N., Lapa J. (2021). The impact of COVID-19 on young people’s mental health in Latvia. Eur. Psychiatry.

[57] (2022). ATC/DDD Index. World Health Organization. https://www.whocc.no/atc_ddd_index/.

[58] McGill M.R., Jaeschke H. (2013). Metabolism and Disposition of Acetaminophen: Recent Advances in Relation to Hepatotoxicity and Diagnosis. Pharm. Res..

[59] King D.H. (1988). History, pharmacokinetics, and pharmacology of acyclovir. J. Am. Acad. Dermatol..

[60] Rosenzweig P., Canal M., Patat A., Bergougnan L., Zieleniuk I., Bianchetti G. (2002). A review of the pharmacokinetics, tolerability and pharmacodynamics of amisulpride in healthy volunteers. Hum. Psychopharmacol. Clin. Exp..

[61] Kuyper L.M., Khan N.A. (2014). Atenolol vs Nonatenolol β-Blockers for the Treatment of Hypertension: A Meta-analysis. Can. J. Cardiol..

[62] Lennernäs H. (2003). Clinical Pharmacokinetics of Atorvastatin. Clin. Pharmacokinet..

[63] Thomaidis N.S., Gago-Ferrero P., Ort C., Maragou N.C., Alygizakis N.A., Borova V.L., Dasenaki M.E. (2016). Reflection of Socioeconomic Changes in Wastewater: Licit and Illicit Drug Use Patterns. Environ. Sci. Technol..

[64] Lancaster S.G., Sorkin E.M. (1988). Bisoprolol. Drugs.

[65] Rice J., Kannan A.M., Castrignanò E., Jagadeesan K., Kasprzyk-Hordern B. (2020). Wastewater-based epidemiology combined with local prescription analysis as a tool for temporalmonitoring of drugs trends—A UK perspective. Sci. Total Environ..

[66] Bahlmann A., Brack W., Schneider R.J., Krauss M. (2014). Carbamazepine and its metabolites in wastewater: Analytical pitfalls and occurrence in Germany and Portugal. Water Res..

[67] Eichelbaum M., Köthe K.W., Hoffmann F., von Unruh G.E. (1979). Kinetics and metabolism of carbamazepine during combined antiepileptic drug therapy. Clin. Pharmacol. Ther..

[68] Borner K., Höffken G., Lode H., Koeppe P., Prinzing C., Glatzelc P., Wiley R., Olschewski P., Sievers B., Reinitz D. (1986). Pharmacokinetics of ciprofloxacin in healthy volunteers after oral and intravenous administration. Eur. J. Clin. Microbiol. Infect. Dis..

[69] Drew R.H., Gallis H.A. (1992). Azithromycin: Spectrum of activity. pharmacokinetics, and clinical applications. Pharmacotherapy.

[70] Benowitz N.L., Hukkanen J., Jacob P. (2009). Nicotine chemistry, metabolism, kinetics and biomarkers. Handb. Exp. Pharmacol..

[71] Stierlin H., Faigle J.W. (1979). Biotransformation of Diclofenac Sodium (Voltaren^®^) in Animals and in Man.: II. Quantitative determination of the unchanged drug and principal phenolic metabolites, in urine and bile. Xenobiotica.

[72] Edmunds M.W., Mayhew M.S. (2009). Chapter 61: Macrolides. Pharmacology for the Primary Care Provider.

[73] Kirst H.A., Sides G.D., Bryskier A., Butzler J.P., Neu H.C., Tulkens (1993). Chapter 28: Erythromycin. The Macrolides.

[74] López-García E., Pérez-López C., Postigo C., Andreu V., Bijlsma L., González-Mariño I., Hernández F., Marcé R.M., Montes R., Picó Y. (2020). Assessing alcohol consumption through wastewater-based epidemiology: Spain as a case study. Drug Alcohol Depend..

[75] Bockbrader H.N., Wesche D., Miller R., Chapel S., Janiczek N., Burger P. (2010). A Comparison of the Pharmacokinetics and Pharmacodynamics of Pregabalin and Gabapentin. Clin. Pharmacokinet..

[76] Evans A.M., Nation R.L., Sansom L.N., Bochner F., Somogyi A.A. (1990). The relationship between the pharmacokinetics of ibuprofen enantiomers and the dose of racemic ibuprofen in humans. Biopharm. Drug Dispos..

[77] Weber M., Kodjikian L., Kruse F.E., Zagorski Z., Allaire C.M. (2012). Efficacy and safety of indomethacin 0.1% eye drops compared with ketorolac 0.5% eye drops in the management of ocular inflammation after cataract surgery. Acta Ophthalmol..

[78] Avapro (Irbesartan) Drug Description. https://www.rxlist.com/avapro-drug.htm#description.

[79] Ketoprofen Drug Description. https://www.drugs.com/pro/ketoprofen.html.

[80] Highlights of Prescribing Information. Levetiracetam. https://www.accessdata.fda.gov/drugsatfda_docs/label/2017/021035s100.021505s040lbl.pdf.

[81] Israili Z.H. (2000). Clinical pharmacokinetics of angiotensin II (AT1) receptor blockers in hypertension. J. Hum. Hypertens..

[82] Dunn C.J., Peters D.H. (1995). Metformin. Drugs.

[83] Metoprolol Succinate. Extended-Release Tablets. https://www.accessdata.fda.gov/drugsatfda_docs/label/2008/019962s036lbl.pdf.

[84] Falany C.N., Ström P., Swedmark S. (2005). Sulphation of o-desmethylnaproxen and related compounds by human cytosolic sulfotransferases. Br. J. Clin. Pharmacol..

[85] EFFEXOR XR—Venlafaxine Hydrochloride Capsule. Extended Release. https://www.accessdata.fda.gov/drugsatfda_docs/label/2008/020699s081lbl.pdf.

[86] Al-Omar M.A. (2009). Chapter 6 ofloxacin. Profiles Drug Subst. Excip. Relat. Methodol..

[87] DailyMed Label: TRILEPTAL (Oxcarbazepine) Film-Coated Tablets or Suspension. for Oral Use. https://dailymed.nlm.nih.gov/dailymed/drugInfo.cfm?setid=4c5c86c8-ab7f-4fcf-bc1b-5a0b1fd0691b.

[88] Highlights of Prescribing Information. Altace (Ramipril). https://www.accessdata.fda.gov/drugsatfda_docs/label/2013/019901s060lbl.pdf.

[89] Martin P.D., Warwick M.J., Dane A.L., Hill S.J., Giles P.B., Phillips P.J., Lenz E. (2003). Metabolism, Excretion, and pharmacokinetics of rosuvastatin in healthy adult male volunteers. Clin. Ther..

[90] Product Information—Ventolin (Salbutamol). https://s3-us-west-2.amazonaws.com/drugbank/cite_this/attachments/files/000/003/265/original/ventolin_cfc_free_inhaler_pi_004_approved.pdf?1548976568.

[91] Hanyok J.J. (1993). Clinical pharmacokinetics of sotalol. Am. J. Cardiol..

[92] FDA Approved Drug Products: Bactrim (Sulfamethoxazole/Trimethoprim) Oral Tablets. https://www.accessdata.fda.gov/drugsatfda_docs/label/2014/017377s074lbl.pdf.

[93] Dina R., Jafari M. (2000). Angiotensin II-receptor antagonists: An overview. Am. J. Health Pharm..

[94] Vazzana M., Andreani T., Fangueiro J., Faggio C., Silva C., Santini A., Garcia M., Silva A., Souto E. (2015). Tramadol hydrochloride: Pharmacokinetics, pharmacodynamics, adverse side effects, co-administration of drugs and new drug delivery systems. Biomed. Pharmacother..

[95] Product Monograph. Trimethoprim. https://pdf.hres.ca/dpd_pm/00025165.PDF.

[96] Drug Description. Valsartan. https://reference.medscape.com/drug/diovan-valsartan-342325#10.

[97] Plumlee K.H. (2004). Clinical Veterinary Toxicology.

