# Supplementary material for: Pharmaceutical and Recreational Drug Usage Patterns during and Post COVID-19 Determined by Wastewater-Based Epidemiology
Source: Int J Environ Res Public Health. 2024 Feb 9;21(2):206. doi: 10.3390/ijerph21020206 (PMC10888181; doi:10.3390/ijerph21020206)
Supplement: Supplementary file 1 [file ijerph-21-00206-s001.zip › ijerph-2786267-supplementary.pdf]

Supplementary Information for

Pharmaceutical and Recreational Drug Usage Patterns during and Post COVID-19 Determined by Wastewater-Based Epidemiology

Laura Elina Tomsone[a], Romans Neilands[b], Kristina Kokina[b], Vadims Bartkevics[a], Iveta Pugajeva[a*]

[a]*Institute of Food Safety, Animal Health and Environment "BIOR", Lejupes Street 3, Riga LV-1076, Latvia*

[b]*Riga Technical University, Faculty of Natural Sciences and Technology, Kipsalas Street 6B, Riga, LV–1048, Latvia*

Table S1. Parameters used for consumption calculation

Compound	Excretion factor from the literature	Reference	Excretion factor used for calculations	Correction factor	DDD, g [1]
Acetaminophen	0.5-0.7	[2]	0.6		3
ACYCLOVIR	0.90-0.92	[3]	0.91		4
Amisulpride	0.492-0.551	[4]	0.52		0.4
Atenolol	0.85-0.95[a]	[5]	0.9		0.075
Atorvastatin	0.02[b]	[6]	0.02		0.02
Azithromycin	0.12	[7]	0.09		0.5
Bisoprolol	0.5	[8]	0.5		0.01
Caffeine	0.046	[9]	0.046		
Carbamazepine	0.12[a]	[10]	0.12		1
Carbamazepine-10,11-epoxide	0.0114	[11]	0.017	0.94	1
Ciprofloxacin	0.27	[12]	0.27		1
Clarithromycin	0.2	[13]	0.2		0.5
Cotinine	0.22-0.32[a]	[14]	0.27	0.92	
Diclofenac	0.048-0.115[a]	[15]	0.082		0.1
Erythromycin	0.02-0.15	[16]	0.085		1
Ethyl sulphate	0.00012	[17]	0.00012	0.37	
Gabapentin	0.99	[18]	0.99		1.8
Ibuprofen	0.12[a]	[19]	0.12[a]		1.2
Indomethacin	0.26[a]	[20]	0.26		0.1
Irbesartan	0.2	[21]	0.2		0.15
Ketoprofen	0.8a	[22]	0.8		0.15
Levetiracetam	0.66	[23]	0.66		1.5
Losartan	0.04-0.05[b]	[24]	0.045		0.05
Metformin	0.9	[25]	0.9		2
Metoprolol	0.05-0.10	[26]	0.075		0.15
Naproxen	0.66-0.92	[27]	0.79		0.5
O-desmethyl venlafaxine	0.55	[28]	0.55	1.05	0.1
Ofloxacin	0.9	[29]	0.9		0.4
Oxcarbazepine	0.13[a]	[30]	0.13		1
Ramipril	0.02	[31]	0.02		0.0025
Rosuvastatin	0.768	[32]	0.768		0.01
Salbutamol	0.6-0.7	[33]	0.65		0.01
Sotalol	0.8-0.9	[34]	0.85		0.16
Sulfamethoxazole	0.3[b]	[35]	0.3		2
Telmisartan	0.97	[36]	0.97		0.04
Tramadol	0.3	[37]	0.3		0.3
Trimethoprim	0.8	[38]	0.8		0.4
Valsartan	0.8	[39]	0.8		0.08
Venlafaxine	0.05	[28]	0.05		0.1
Xylometazoline	0.5	[40]	0.5		0.0008

[a]Total excretion of biomarker and its glucuronides
[b]Biomarker excretion without glucuronides

1. WHO Collaborating Centre for Drug Statistics Methodology. ATC classification index with DDDs. 2022. Oslo. Norway 2021. https://www.whocc.no/atc_ddd_index/
2. McGill. M.R.; Jaeschke. H.; 2013. Metabolism and Disposition of Acetaminophen: Recent Advances in Relation to Hepatotoxicity and Diagnosis. Pharm. Res. 30. 2174–2187. https://doi.org/10.1007/s11095-013-1007-6
3. King. D.H.; 1988. History. pharmacokinetics. and pharmacology of acyclovir. J. Am. Acad. Dermatol. 18(1 Pt 2):176-9. doi: 10.1016/s0190-9622(88)70022-5.
4. Rosenzweig. P.; Canal. M.; Patat. A.; Bergougnan. L.; Zieleniuk. I. and Bianchetti. G.; 2002. A review of the pharmacokinetics. tolerability and pharmacodynamics of amisulpride in healthy volunteers. Hum. Psychopharmacol. Clin. Exp.; 17: 1-13. https://doi.org/10.1002/hup.320
5. Kuyper. L.M.; Khan. N.A.; 2014. Atenolol vs nonatenolol β-blockers for the treatment of hypertension: a meta-analysis. Can. J. Cardiol. 30(5 Suppl):S47-53. doi: 10.1016/j.cjca.2014.01.006
6. Lennernäs. H.; 2003. Clinical Pharmacokinetics of Atorvastatin. Clin. Pharmacokinet. 42. 1141–1160. https://doi.org/10.2165/00003088-200342130-00005
7. Thomaidis. N.S.; Gago-Ferrero. P.; Ort. C.; Maragou. N.C.; Alygizakis. N.A.; Borova. V.L.; Dasenaki. M.E.; 2016. Reflection of Socioeconomic Changes in Wastewater: Licit and Illicit Drug Use Patterns. Environ. Sci. Technol. 20;50(18):10065-72. doi: 10.1021/acs.est.6b02417
8. Lancaster. S.G.; Sorkin. E.M.; 1988. Bisoprolol. A preliminary review of its pharmacodynamic and pharmacokinetic properties. and therapeutic efficacy in hypertension and angina pectoris. Drugs 36:256-285. 10.2165/00003495-198836030-00002
9. Rice. J.; Kannan. A.M.; Castrignanò. E.; Jagadeesan. K.; Kasprzyk-Hordern. B.; 2020. Wastewater-based epidemiology combined with local prescription analysis as a tool for temporalmonitoring of drugs trends - A UK perspective. Sci. Total Environ. 735:139433
10. Bahlmann. A.; Brack. W.; Schneider. R.J.; Krauss. M.; 2014. Carbamazepine and its metabolites in wastewater: Analytical pitfalls and occurrence in Germany and Portugal. Water Res. 57. 104-114. https://doi.org/10.1016/j.watres.2014.03.022."
11. Eichelbaum. M.; Köthe. K. W.; Hoffmann. F.; and von Unruh. G. E.; 1979. Kinetics and metabolism of carbamazepine during combined antiepileptic drug therapy. Clin. Pharmacol. Ther. 26. doi: 10.1002/cpt1979263366
12. Borner. K.; Höffken. G.; Lode. H.; Koeppe. P.; Prinzing. C.; Glatzel. P.; Wiley. R.; Olschewski. P.; Sievers. B.; Reinitz. D.; 1986. Pharmacokinetics of ciprofloxacin in healthy volunteers after oral and intravenous administration. Eur. J. Clin. Microbiol. 5(2):179-86. doi: 10.1007/BF02013983

13. Drew. R.H.; Gallis. H.A.; 1992. Azithromycin: Spectrum of activity. pharmacokinetics. and clinical applications. Pharmacotherapy. 12:161–73.
14. Benowitz. N.L.; Hukkanen. J.; Jacob. P. 3rd.; 2009. Nicotine chemistry. metabolism. kinetics and biomarkers. Handb. Exp. Pharmacol. (192):29-60. doi: 10.1007/978-3-540-69248-5_2
15. Stierlin. H.; Faigle. J.W.; 1979. Biotransformation of diclofenac sodium (Voltaren) in animals and in man. II. Quantitative determination of the unchanged drug and principal phenolic metabolites. in urine and bile. Xenobiotica. 9(10):611-21. doi: 10.3109/00498257909042328
16. Edmunds. M.W.; Mayhew. M.S.; 2009. "Chapter 61: Macrolides". Pharmacology for the primary care provider (Third ed.). Saint Louis. Missouri. pp. 658–662 (661). ISBN 978-0-323-06316-6.; Kirst HA. Sides GD (1993). "Chapter 28: Erythromycin". In Bryskier A. Butzler JP. Neu HC. Tulkens (eds.). The Macrolides. Oxford UK: Arnette-Blackwell.
17. López-García. E.; Pérez-López. C.; Postigo. C.; Andreu. V.; Bijlsma. L.; González-Mariño. I.; Hernández. F.; Marcé. R.M.; Montes. R.; Picó. Y.; Pocurull. E.; Rico. A.;Rodil. R.; Rosende. M.; Valcárcel. Y.; Zuloaga. O.; Quintana. J.B.; López de Alda. M.; 2020. Assessing alcohol consumption through wastewater-based epidemiology: Spain as a case study. Drug Alcohol Depend. 215.108241. https://doi.org/10.1016/j.drugalcdep.2020.108241.
18. Bockbrader. H.N.; Wesche. D.; Miller. R.; Chapel. S.; Janiczek. N.; Burger. P.; 2010. A comparison of the pharmacokinetics and pharmacodynamics of pregabalin and gabapentin. Clin Pharmacokinet. 49(10):661-9. doi: 10.2165/11536200-000000000-00000
19. Evans. A.M.; Nation. R.L.; Sansom. L.N.; Bochner. F. and Somogyi. A.A.; 1990. The relationship between the pharmacokinetics of ibuprofen enantiomers and the dose of racemic ibuprofen in humans. Biopharmaceutics & drug disposition. 11(6). pp.507-518.
20. Weber. M.; Kodjikian. L.; Kruse. F.E.; Zagorski. Z.; Allaire. C.M.; 2013. Efficacy and safety of indomethacin 0.1% eye drops compared with ketorolac 0.5% eye drops in the management of ocular inflammation after cataract surgery. Acta Ophthalmol. 91(1):e15-21. doi: 10.1111/j.1755-3768.2012.02520.x.
21. Avapro (irbesartan) drug description. https://www.rxlist.com/avapro-drug.htm#description (accessed 1 December 2021)
22. Ketoprofen drug description. https://www.drugs.com/pro/ketoprofen.html (accessed 1 December 2021)
23. Highlights of prescribing information. Levetiracetam. https://www.accessdata.fda.gov/drugsatfda_docs/label/2017/021035s100.021505s040lbl.pdf (accessed 1 December 2021)

24. Israili. Z.H.; 2000. Clinical pharmacokinetics of angiotensin II (AT1) receptor blockers in hypertension. J. Hum. Hypertens. 14 Suppl 1:S73-86. doi: 10.1038/sj.jhh.1000991
25. Dunn. C.J.; Peters. D.H.; 1995. Metformin. A review of its pharmacological properties and therapeutic use in non-insulin-dependent diabetes mellitus. Drugs. 49(5):721-49. doi: 10.2165/00003495-199549050-00007
26. Metoprolol succinate. Extended-release tablets. https://www.accessdata.fda.gov/drugsatfda_docs/label/2008/019962s036lbl.pdf (accessed 1 December 2021)
27. Falany. C.N.; Ström. P.; Swedmark. S.; 2005. Sulphation of o-desmethylnaproxen and related compounds by human cytosolic sulfotransferases. Br. J. Clin. Pharmacol. 60(6):632-40. doi: 10.1111/j.1365-2125.2005.02506.x
28. EFFEXOR XR - venlafaxine hydrochloride capsule. extended release. https://www.accessdata.fda.gov/drugsatfda_docs/label/2008/020699s081lbl.pdf (accessed 1 December 2021)
29. Al-Omar. M. A.; 2009. Ofloxacin. Profiles of Drug Substances. Excipients and Related Methodology. 265–298. doi:10.1016/s1871-5125(09)34006-6
30. DailyMed Label: TRILEPTAL (oxcarbazepine) film-coated tablets or suspension. for oral use. https://dailymed.nlm.nih.gov/dailymed/drugInfo.cfm?setid=4c5c86c8-ab7f-4fcf-bc1b-5a0b1fd0691b (accessed 1 December 2021)
31. Highlights of prescribing information. Altace (ramipril). https://www.accessdata.fda.gov/drugsatfda_docs/label/2013/019901s060lbl.pdf (accessed 1 December 2021)
32. Martin. P.D.; Warwick. M.J.; Dane. A.L.; Hill. S.J.; Giles. P.B.; Phillips. P.J.; Lenz. E.; 2003. Metabolism. excretion. and pharmacokinetics of rosuvastatin in healthy adult male volunteers. Clin. Ther. 25(11):2822-35.
33. Product information - Ventolin (salbutamol). https://s3-us-west-2.amazonaws.com/drugbank/cite_this/attachments/files/000/003/265/original/ventolin_cfc_free_inhaler_pi_004_approved.pdf?1548976568 (accessed 1 December 2021)
34. Hanyok. J.J.; 1993. Clinical pharmacokinetics of sotalol. Am. J. Cardiol. 12;72(4):19A-26A.
35. FDA Approved Drug Products: Bactrim (sulfamethoxazole/trimethoprim) oral tablets. https://www.accessdata.fda.gov/drugsatfda_docs/label/2014/017377s074lbl.pdf (accessed 1 December 2021)
36. Dina. R.; Jafari. M.; 2000. Angiotensin II-Receptor Antagonists: An Overview. Am. J. Syst. Pharm. 57(13) https://doi.org/10.1093/ajhp/57.13.1231

37. Vazzana. M.; Andreani. T.; Fangueiro. J.; Faggio. C.; Silva. C.; Santini. A.; Garcia. M.L.; Silva. A.M.; Souto. E.B.; 2015. Tramadol hydrochloride: pharmacokinetics. pharmacodynamics. adverse side effects. co-administration of drugs and new drug delivery systems. Biomed. Pharmacother. 70:234-8. doi: 10.1016/j.biopha.2015.01.022
38. Product monograph. Trimethoprim. https://pdf.hres.ca/dpd_pm/00025165.PDF (accessed 1 December 2021)
39. Drug description. Valsartan. https://reference.medscape.com/drug/diovan-valsartan-342325#10 (accessede 1 December 2021)
40. Plumlee. K.H.; 2004. Chapter 24 - Pharmaceuticals. Editor(s): Konnie H. Plumlee. Clinical Veterinary Toxicology. Mosby.Pages 282-336.https://doi.org/10.1016/B0-32-301125-X/50027-3."

Table S2. Concentrations and corresponding consumptions of the detected compounds in tested wastewater samples

Therapeutic group	Compound	Concentration, ng L^{-1}			Average consumption, mg 1000 inh^{-1} day^{-1}	Average consumption, DDD 1000 inh^{-1} day^{-1}
		Range	Median	Average		
Macrolide antibiotic	Azithromycin	67-1,727	515	540	1,550	3.1
	Clarithromycin	66-1,361	499	507	872	1.7
	Erythromycin	4.1-210	69	79	527	0.53
Fluoroquinolone antibiotic	Ciprofloxacin	65-766	250	281	356	0.36
	Ofloxacin	8.2-124	30	33	13	0.032
Sulphanilamide antibiotic	Sulfamethoxazole	128-1,544	631	693	787	0.39
	Trimethoprim	16-348	146	149	64	0.16
NSAID	Diclofenac	392-4,190	1,897	1,997	9,539	95
	Ibuprofen	2,914-14,831	10,886	10,735	29,731	25
	Indomethacin	9.0-229	44	48	61	0.61
	Ketoprofen	77-955	482	483	180	1.2
	Naproxen	49-3,758	835	966	444	0.89
Antiepileptic	Carbamazepine	141-922	508	510	1,462	1.5
	Carbamazepine-10,11-epoxide[a]	8.8-89	44	45	1,253	1.3
	Gabapentin	3743-34,969	15,068	15,240	5,116	2.8
	Levetiracetam	637-4,302	1,980	2,075	1,053	0.70
	Oxcarbazepine	17-1,086	168	208	550	0.55
ACE inhibitor	Ramipril	1.3-15	5.8	6.9	114	46
Angiotensin II receptor blocker	Irbesartan	0.92-32	11	12	20	0.13
	Losartan	33-339	170	176	1,480	30
	Telmisartan	14-3,233	1,312	1,305	455	11
	Valsartan	285-4,002	1,820	1,953	816	10
Beta-blocker	Atenolol	2.6-73	23	29	11	0.14
	Bisoprolol	66-559	302	309	207	21
	Metoprolol	44-589	263	274	1,330	8.9
	Sotalol	128-631	419	411	163	1.0
Analgesic	Acetaminophen	6,411-35,141	17,624	18,379	10,346	3.4
Antiasthmatic	Salbutamol	2.4-28	8.3	9.5	5.3	0.52
Antidepressant	O-desmethyl venlafaxine[b]	86-771	334	339	220	2.2
	Venlafaxine	6.2-132	50	52	353	3.5
Antidiabetic	Metformin	19,687-127,079	81,384	82,758	30,414	15
Antihyperlipidemic	Atorvastatin	30-970	215	225	757	38
	Rosuvastatin	368-3,002	1,414	1,458	647	65
Antiviral	Acyclovir	593-3,731	1,850	1,858	689	0.17
Decongestant	Xylometazoline	1.4-35	8.6	11	7.1	8.8
Opioid	Tramadol	49-663	231	251	281	0.94
Psychiatric	Amisulpride	33-255	126	132	89	0.22
Recreational	Ethyl sulphate[c]	6,102-42,034	20,222	18,707	21,100	
	Caffeine	15,774-107,708	65,392	66,215	470,671	
	Cotinine[d]	845-6,453	4,246	4,063	4,575	

[a] Consumption represents carbamazepine
[b] Consumption represents venlafaxine
[c] Consumption represents alcohol
[d] Consumption represents nicotine

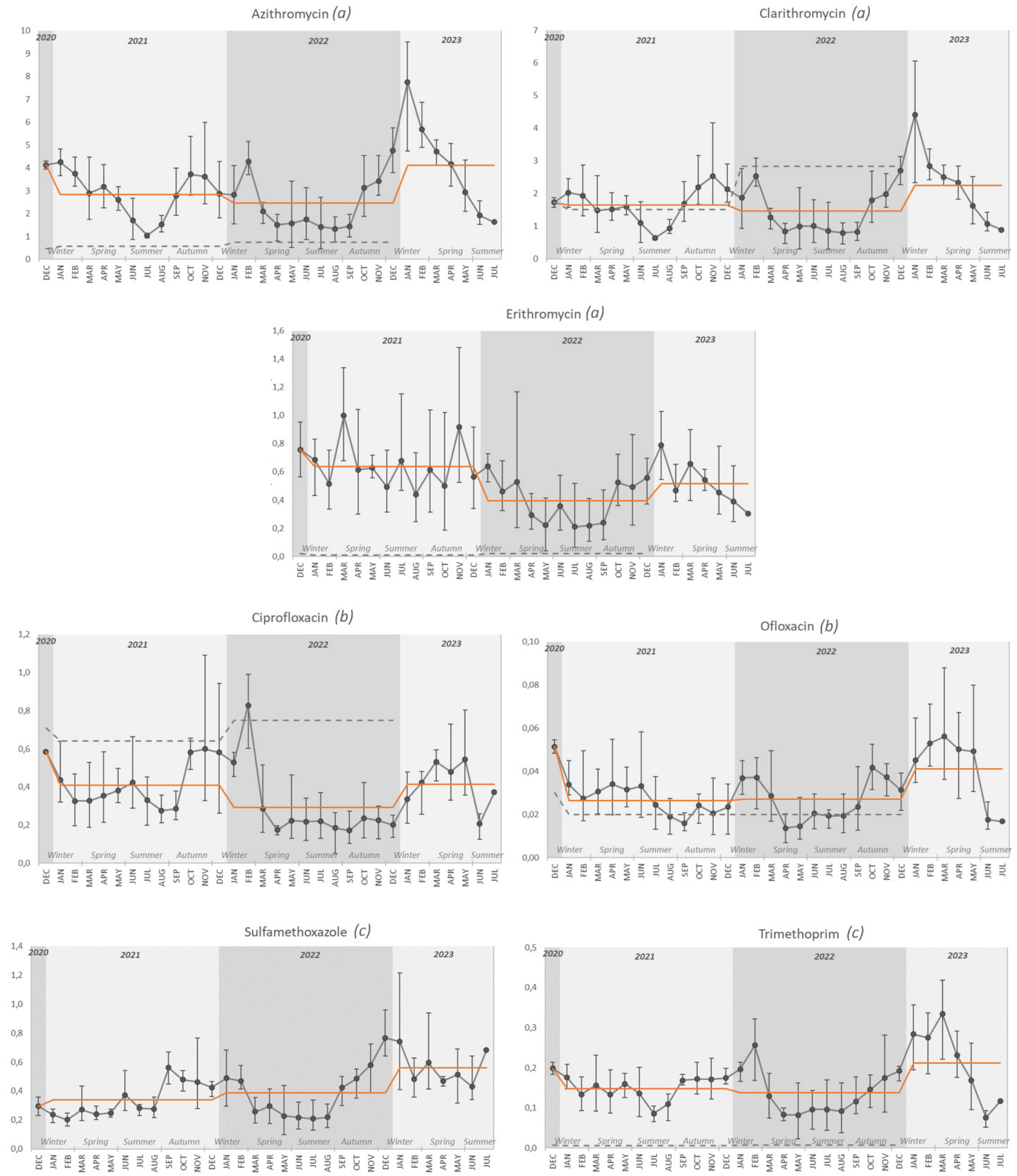

Figure S1. Seasonal variations of antibiotic consumption (DDD 1000 inh^{-1} day^{-1}): a) macrolides; b) fluoroquinolones, and c) sulphanilamides (error bar shows range of detection in each month, orange line marks the yearly average consumption, and grey dashed line represents consumption data from sales statistics)

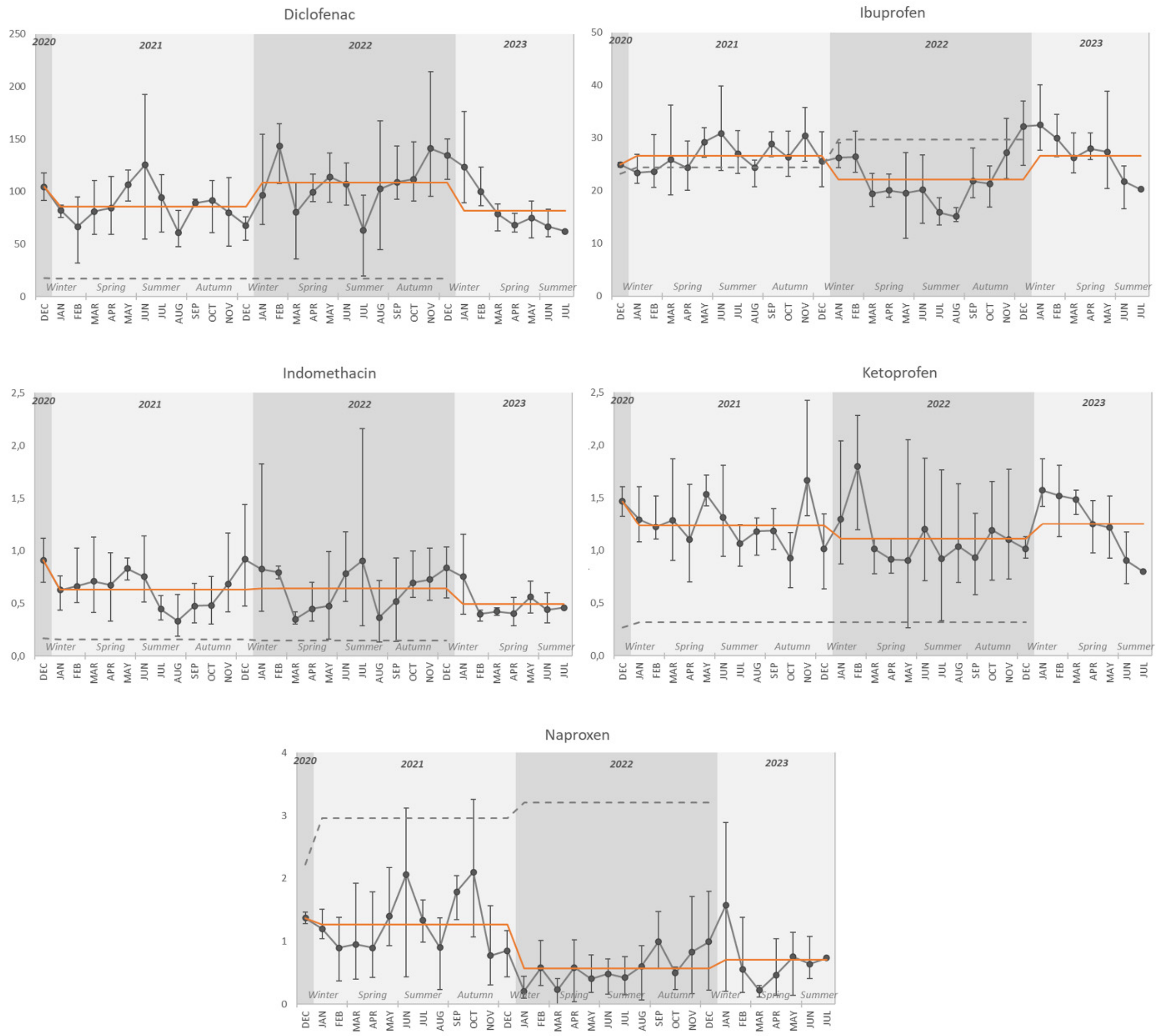

Figure S2. Seasonal variations of NSAID consumption (DDD 1000 inh^{-1} day^{-1}; error bar shows range of detection in each month, orange line marks the yearly average consumption, and grey dashed line represents consumption data from sales statistics)

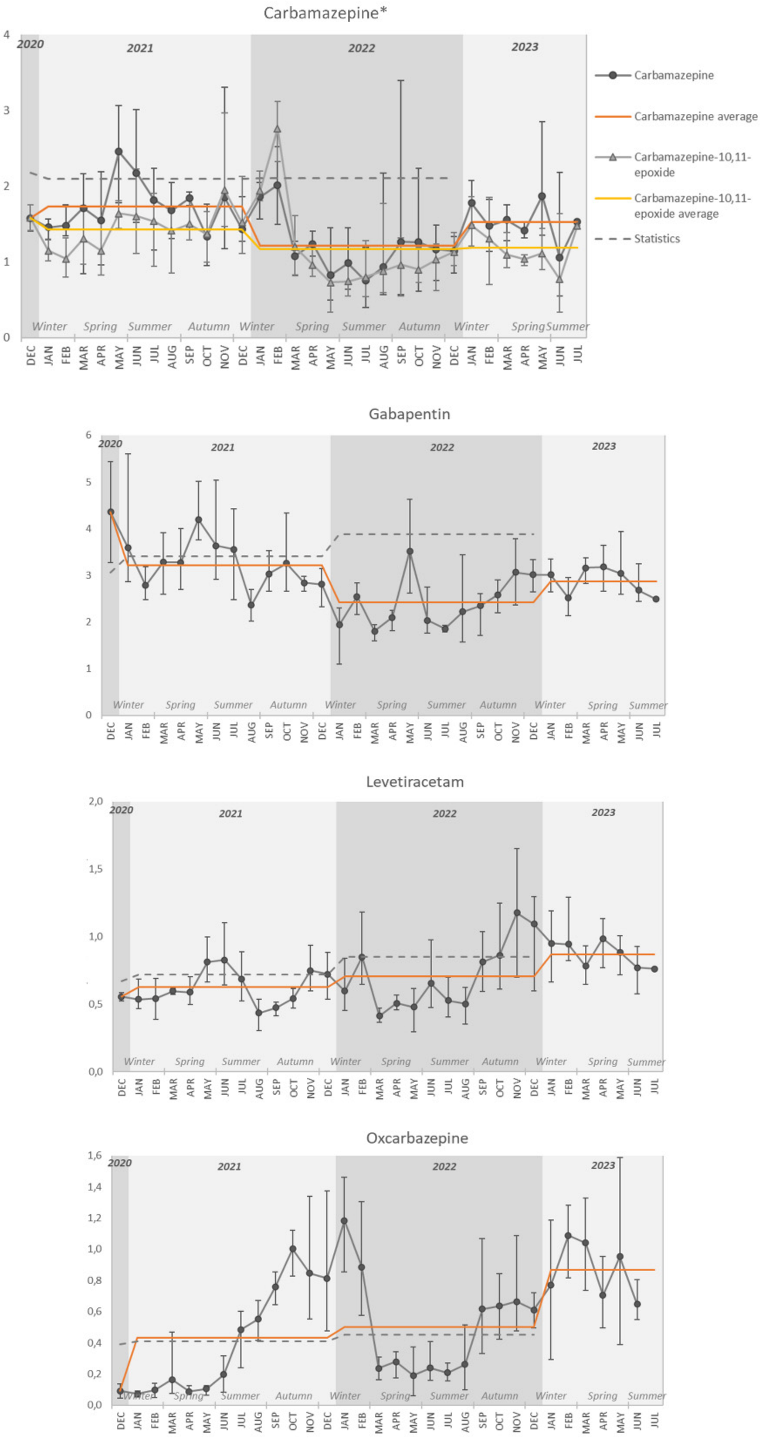

Figure S3. Seasonal variations of antiepileptic consumption (DDD 1000 inh^{-1} day^{-1}; error bar shows range of detection in each month, orange line marks the yearly average consumption, and grey dashed line represents consumption data from sales statistics; carbamazepine consumption obtained both from parent compound and metabolite is shown)

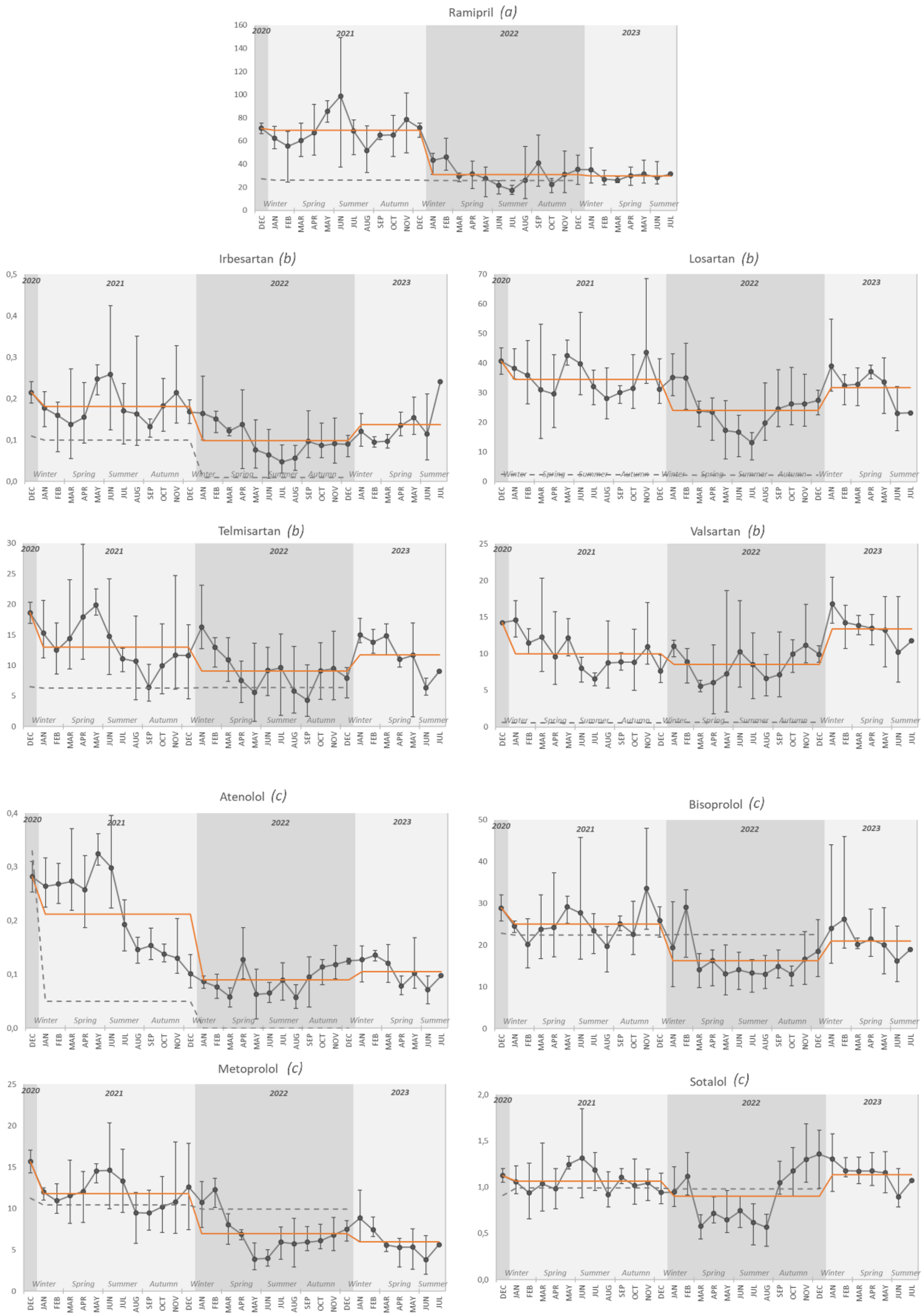

Figure S4. Seasonal variations of antiepileptic consumption (DDD 1000 inh^{-1} day^{-1}): a) ACE inhibitor; b) angiotensin II receptor blockers, and c) beta-blockers (error bar shows range of detection in each month, orange line marks the yearly average consumption, and grey dashed line represents consumption data from sales statistics)

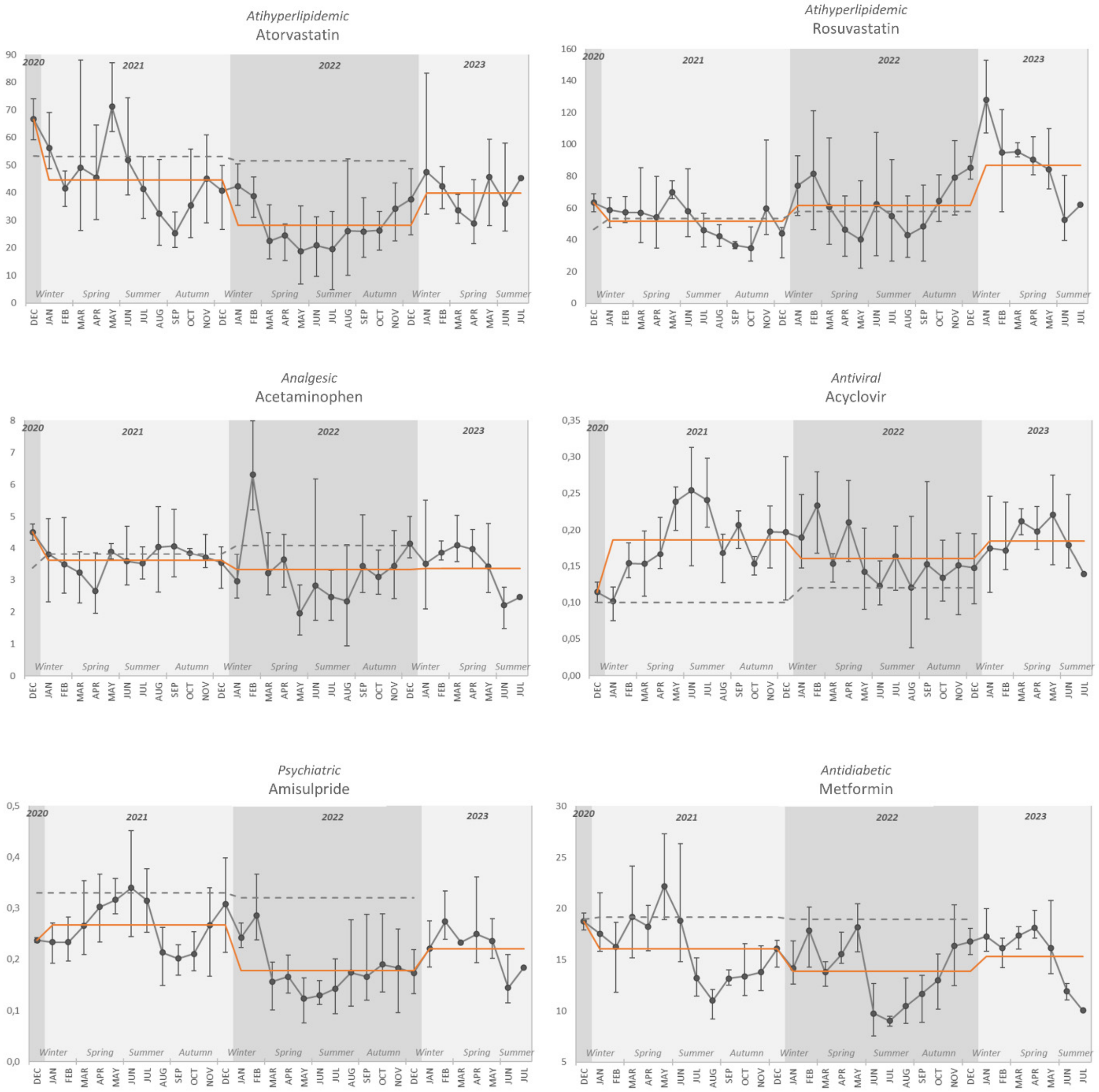

Figure S5. Seasonal variations of the consumption of other pharmaceuticals included in the study (DDD 1000 inh^{-1} day^{-1}; error bar shows range of detection in each month, orange line marks the yearly average consumption, and grey dashed line represents consumption data from sales statistics)

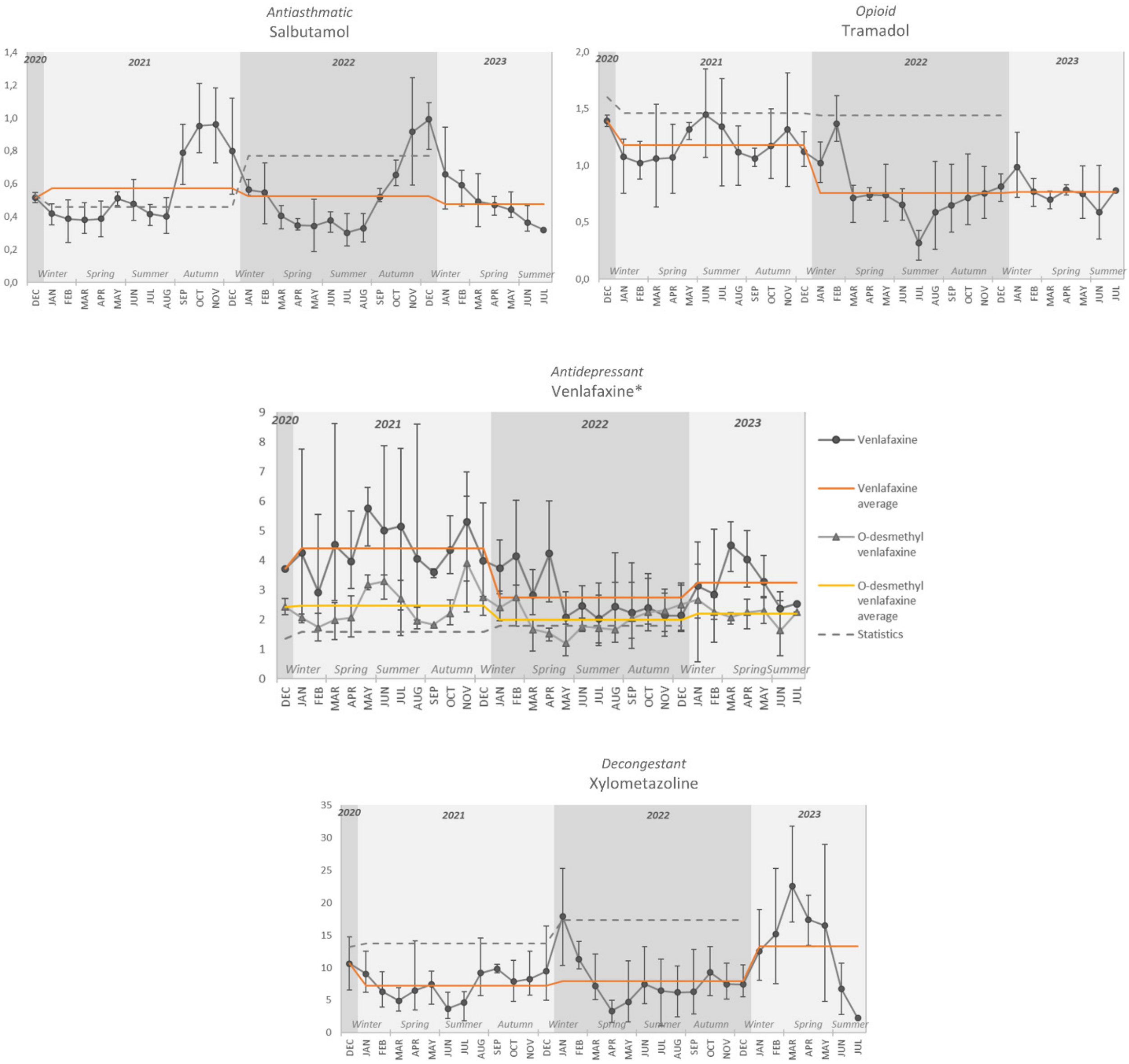

Figure S6. Seasonal variations of the consumption of other pharmaceuticals included in the study (DDD 1000 inh^{-1} day^{-1}; error bar shows range of detection in each month, orange line marks the yearly average consumption, and grey dashed line represents consumption data from sales statistics; venlafaxine consumption obtained both from parent compound and metabolite is shown)
